# The calcium sensor Copine-6 regulates spine structural plasticity and learning and memory

**DOI:** 10.1038/ncomms11613

**Published:** 2016-05-19

**Authors:** Judith R. Reinhard, Alexander Kriz, Milos Galic, Nico Angliker, Mathieu Rajalu, Kaspar E. Vogt, Markus A. Ruegg

**Affiliations:** 1 Biozentrum, University of Basel, Klingelbergstrasse 70, 4056 Basel, Switzerland

## Abstract

Hippocampal long-term potentiation (LTP) represents the cellular response of excitatory synapses to specific patterns of high neuronal activity and is required for learning and memory. Here we identify a mechanism that requires the calcium-binding protein Copine-6 to translate the initial calcium signals into changes in spine structure. We show that Copine-6 is recruited from the cytosol of dendrites to postsynaptic spine membranes by calcium transients that precede LTP. *Cpne6* knockout mice are deficient in hippocampal LTP, learning and memory. Hippocampal neurons from *Cpne6* knockouts lack spine structural plasticity as do wild-type neurons that express a Copine-6 calcium mutant. The function of Copine-6 is based on its binding, activating and recruiting the Rho GTPase Rac1 to cell membranes. Consistent with this function, the LTP deficit of *Cpne6* knockout mice is rescued by the actin stabilizer jasplakinolide. These data show that Copine-6 links activity-triggered calcium signals to spine structural plasticity necessary for learning and memory.

## Abstract (web summary)

Learning and memory depend on the structural and functional plasticity of synapses. Reinhard *et al*. show that the calcium sensor Copine-6 is required for memory, controls spine structure by regulating Rac signalling, and through its modulation of actin, supports hippocampal LTP.

## Graphical abstract

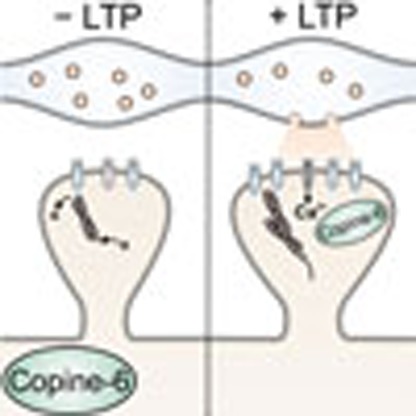

Long-term changes in synaptic efficacy are thought to be critical for learning and memory^1^,^2^,^3^,^4^. Long-term potentiation (LTP) and long-term depression (LTD) are well accepted to be cellular correlates of the change in synaptic efficacy. NMDA (*N*-methyl-D-aspartate) receptor-dependent LTP in the hippocampus is the best studied form of this change in synaptic efficacy. It is initiated by high neuronal activity that triggers the opening of NMDA receptors and thereby increases calcium influx into postsynaptic spines, which are small, actin-rich protrusions of dendrites^5^,^6^. Increase in intracellular calcium then triggers an intracellular signalling cascade that results in the subsequent recruitment of AMPA (α-amino-3-hydroxy-5-methyl-4-isoxazole propionic acid) receptors to the spines and their local incorporation into the postsynaptic membrane^7^. This recruitment of AMPA receptors is regulated by changes in the actin cytoskeleton dynamics and has been shown to correlate with the increase in the size of the postsynaptic spines^8^,^9^. Calcium/calmodulin-dependent kinase II (CaMKII) strongly contributes to the early phase of LTP through its interaction with the NMDA receptors^10^. CaMKII has also been implicated in the later phase of LTP but the precise mechanisms of how it affects actin remodelling remain elusive^10^.

Copines form a small family of cytosolic proteins that are characterized by two C2 domains, known to bind phospholipids in a calcium-dependent manner^11^, and an A domain at the carboxy terminus. Copines are evolutionary conserved and are expressed from *Paramecium* to humans^12^. Some Copines have been shown to translocate to plasma membranes on calcium influx when overexpressed in heterologous cells^13^,^14^,^15^. However, the function of Copines is not well defined in any species, except *Caenorhabditis elegans* where one Copine is required for the surface targeting and stabilization of neurotransmitter receptors at the plasma membrane^16^. Sequence identity and domain structure predicts that the mammalian genome codes for nine Copines. Most of them are expressed ubiquitously. One of the exceptions is Copine-6, whose expression is restricted to the brain. In hippocampal neurons, *Cpne6* expression is upregulated by experimental induction of brief seizures or after induction of LTP^17^. Furthermore, proteomic analyses have shown that Copine-6 is present in postsynaptic densities (PSDs)^18^,^19^.

Here we investigated the function of Copine-6 in the mouse brain. We find that *Cpne6* transcripts and Copine-6 protein are expressed in the postnatal brain with peak expression in the hippocampus. Calcium transients triggered by chemical LTP (cLTP) cause the translocation of Copine-6 from the dendrite to postsynaptic spine membranes. Importantly, *Cpne6* knockout (KO) mice are impaired in hippocampal LTP and in hippocampus-dependent learning and memory. Copine-6 binds to the Rho GTPase Rac1 and recruits Rac1 to plasma membranes in response to calcium influx in heterologous cells. LTP-inducing paradigms applied to *Cpne6* KO neurons or to neurons that express a calcium mutant of Copine-6 do not enrich Rac1 or its target Cofilin in spines and do not cause spine enlargement. Finally, the LTP-deficit in *Cpne6* KO hippocampi is restored by jasplakinolide, a pharmacological agent that stabilizes actin filaments. In summary, these data establish Copine-6 as a critical component in the mouse hippocampus to link activity-triggered calcium signals to spine structural plasticity, learning and memory.

## Results

### Copine-6 is expressed in postnatal excitatory neurons

**Figure 1:**
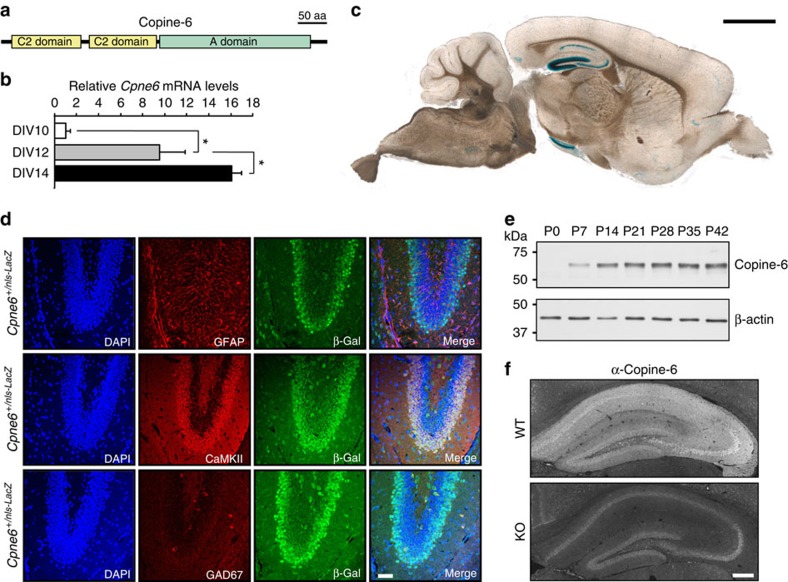
Expression of Copine-6 in cultured hippocampal neurons and postnatal brain. (**a**) Schematic illustration of the domain architecture of Copine-6. (**b**) *Cpne6* mRNA levels were measured in low-density primary rat hippocampal cultures by real-time PCR at DIV 10, 12 and 14. Data are mean±s.d. from *n*=2 cultures per time point. *P<0.05 by one-way ANOVA with Tukey's *post-hoc* test. (**c**) Sagittal brain section of a 6-week-old *Cpne6*^*+/nls-LacZ*^ mouse. *Cpne6*-driven nls-LacZ expression is detected by the blue β-Gal staining in the nuclei. Scale bar, 1.5 mm. (**d**) Coronal sections of the dentate gyrus from 2-week-old *Cpne6*^*+/nls-LacZ*^ mice were stained with DAPI (4,6-diamidino-2-phenylindole; blue), antibodies to β-galactosidase (β-Gal; green) and the antigens indicated. Glial fibrillary acidic protein (GFAP; astrocyte marker); calcium/calmodulin-dependent protein kinase II (CaMKII; excitatory neurons); glutamate decarboxylase 67 (GAD67; inhibitory neurons). Scale bar, 50 μm. (**e**) Western blot analysis of hippocampal lysates of mice of indicated age. β-actin was used as loading control. (**f**) Coronal sections of the hippocampus from 6-week-old WT or *Cpne6* KO mice stained with anti-Copine-6 antibodies. Scale bar, 200 μm.

The presence of two C2 domains and one A domain characterizes all the mammalian Copines including Copine-6 (Fig. 1a). Out of the nine Copines identified in mice, *Cpne4*, -*5*, *-6* and -*9* are preferentially expressed in the brain^12^,^20^,^21^. Of those, *Cpne6* seems to be strongly expressed in the adult mouse hippocampus^22^,^23^. To examine the temporal expression pattern of *Cpne6*, we first used low-density, glia-free cultures of hippocampal neurons and tested *Cpne6* messenger RNA (mRNA) expression by real-time PCR between day *in vitro* (DIV) 10 to DIV14, which reflects the time when synapses are formed and consolidated in these cultures^24^. *Cpne6* mRNA, normalized to DIV10, increased steeply at DIV12 and DIV14 (Fig. 1b). The spatial expression of *Cpne6* transcripts in the adult brain was assessed in mice where a reporter cassette encoding β-galactosidase, preceded by a nuclear localization signal (nls-LacZ), was knocked into the *Cpne6* locus (Supplementary Fig. 1a). Staining for β-galactosidase in mice heterozygous for this knock-in allele was mainly confined to the dentate gyrus and the CA regions in adult mouse brain with some staining in the cerebral cortex and the amygdala (Fig. 1c and Supplementary Fig. 1b). To define the cell types that express *Cpne6*, we also co-stained coronal sections of the dentate gyrus for β-galactosidase and markers for glial cells (glial cell fibrillary protein (GFAP)), excitatory neurons (CaMKII) or inhibitory neurons (GAD67). Whereas cells stained for β-galactosidase were not positive for GFAP or GAD67, they were CaMKII positive (Fig. 1d), indicating that *Cpne6* expression is confined to excitatory neurons. Western blot analysis in hippocampal lysates revealed that Copine-6 was not detected at birth, that levels increased between postnatal day 7 (P7) and P28 and then remained high (Fig. 1e). Staining of coronal sections with anti-Copine-6 antibodies revealed strong immunoreactivity in the neuropil of the dentate gyrus and the CA regions (Fig. 1f). In conclusion, these experiments show that Copine-6 is a cytosolic protein that is most strongly expressed in excitatory neurons of the hippocampus. Thus, these experiments suggest that Copine-6 might be involved in a late stage of synapse formation or in the maintenance/plasticity of synapses in pyramidal neurons of the hippocampus.

### Copine-6 responds to calcium influx via NMDA receptors

**Figure 2:**
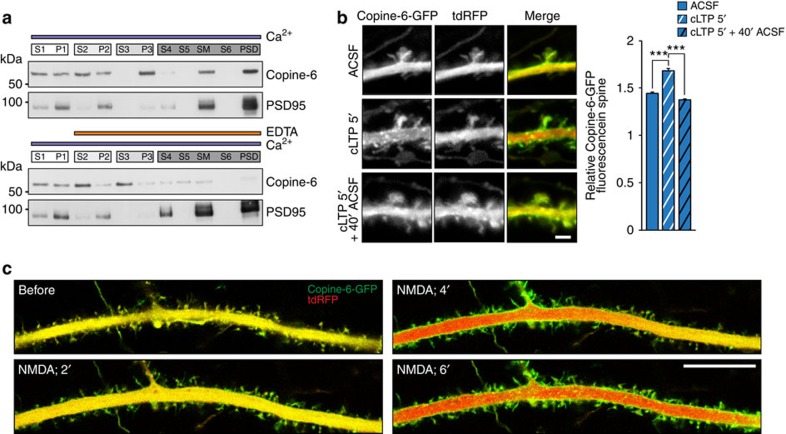
Copine-6 accumulates in spines on NMDA-mediated calcium influx. (**a**) Subcellular fractionation of adult rat brain in the presence of calcium (top) or EDTA, added after the first centrifugation step (bottom). P1–P3, pellets; PSD, postsynaptic densities; S1–S6, supernatants; SM, synaptic membranes (see Methods for details). (**b**) Representative pictures (left) and quantification (right) of DIV21 cultured hippocampal neurons transfected at DIV14 to express Copine-6-GFP and tdRFP after the indicated treatment. For quantification, the ratio of Copine-6-GFP fluorescence in the spine to that in the dendrite was normalized to spine volume (see Methods for details). Copine-6-GFP becomes enriched in spines after 5 min of cLTP (cLTP 5′). The accumulation of Copine-6-GFP is lost after an additional 40 min in ACSF (cLTP 5′+40′ ACSF). Data are mean±s.e.m. from *n*=500 spines per condition (25 spines per neuron from 20 neurons of each condition; from 3 independent cultures). ***P<0.001 by one-way ANOVA with Tukey's *post-hoc* test. Scale bar, 2 μm. (**c**) Time-lapse confocal pictures of DIV14 hippocampal neurons expressing Copine-6-GFP (green) and cytosolic tdRFP (red) during stimulation with 10 μM NMDA. Time points indicate minutes after NMDA application. Scale bar, 20 μm.

Copine-6 contains two C2 domains, which are well known to mediate calcium-dependent binding to phospholipids^11^, and several Copines, when transfected into HEK293 cells, have been shown to associate with the plasma membrane on treatment with the calcium ionophore ionomycin^15^. We therefore hypothesized that Copine-6 might also be membrane-associated in a calcium-dependent manner in neurons. To test this, we purified PSDs from adult rat brains by differential centrifugation in the presence of calcium or after calcium chelation with EDTA. Fractions were tested by western blot analysis using anti-Copine-6 and anti-PSD95 antibodies. In the presence of calcium, Copine-6 co-purified with the PSD marker PSD95, whereas depletion of calcium by EDTA resulted in a cytosolic localization of Copine-6 in fractions S2 and S3 (Fig. 2a). To get an estimate for the calcium concentration needed to cause the binding of Copine-6 to cell membranes, we transfected COS7 cells with expression plasmids encoding green fluorescent protein (GFP)-tagged Copine-6 (Copine-6-GFP). Cells were lysed and separated into cytosolic and membrane fractions in the presence of increasing concentrations of calcium or EGTA, respectively. In this assay, apparent half maximal binding of Copine-6 to plasma membranes was between 1 and 5 μM calcium and depletion of calcium by EGTA released Copine-6 from the membranes (Supplementary Fig. 2a). On the cellular level, the calcium ionophore ionomycin caused the redistribution of Copine-6-GFP from the cytosol to patch-like clusters at the plasma membrane, which were positive for the lipid raft marker choleratoxin B1 (Supplementary Fig. 2b). Disruption of lipid rafts by cholesterol depletion with methyl-β-cyclodextrin significantly inhibited this calcium-mediated association of Copine-6-GFP to membranes (Supplementary Fig. 2c). Finally, fractionation of rat brain lysates in the presence of calcium showed that endogenous Copine-6 co-fractionated with the PSD marker NMDA receptor subunit 1 (NR1) and the lipid raft marker flotilin-1 (Supplementary Fig. 2d). Together, these results provide evidence for a calcium-dependent association of Copine-6 with PSD membranes. They further indicate that this association requires only micromolar concentrations of calcium and that Copine-6 binds preferentially to lipid raft-enriched membranes. Interestingly, PSD membranes are particularly rich in lipid rafts to limit diffusion of neurotransmitter receptors^25^,^26^.

Changes in intracellular calcium are known to trigger synaptic strengthening of excitatory synapses during synaptic plasticity^5^,^27^. To test whether Copine-6 would respond to such synaptic calcium signals, we treated DIV21 hippocampal neurons with medium containing glycine. Such treatment has been shown to trigger cLTP that reproduces NMDA receptor-dependent LTP induced by electrical stimulation^28^,^29^,^30^. To visualize Copine-6 and the overall morphology of the neurons, cells were co-transfected with Copine-6-GFP and the cytosolic volume marker tandem-dimer red fluorescent protein (tdRFP) at DIV14. Incubation of the neurons at DIV21 with cLTP-inducing medium for 5 min was sufficient to cause the accumulation of Copine-6-GFP in spines (Fig. 2b). Interestingly, spine accumulation of Copine-6-GFP was completely lost 40 min after the replacement of the cLTP-inducing solution by artificial cerebrospinal fluid (ACSF) (Fig. 2b).

To get spatio-temporal insights, we used life microscopy of DIV14 neurons in which calcium transients were triggered by NMDA. Only few minutes after NMDA addition, Copine-6-GFP had moved into dendritic protrusions (Fig. 2c and Supplementary Movie 1), whereas the same treatment did not at all change the cytosolic distribution of GFP (Supplementary Movie 2). Quantification before and 4 min after NMDA application showed that Copine-6-GFP was significantly enriched in spines (Supplementary Fig. 3a). A very similar and even faster translocation of Copine-6 to spines was observed by adding glutamate (Supplementary Fig. 3b). This glutamate-induced translocation of Copine-6 was prevented by the NMDA receptor antagonist AP5 (Supplementary Fig. 3c) and the NMDA receptor agonist *trans*-ACBD was sufficient to trigger Copine-6 translocation (Supplementary Fig. 3c). Moreover, blocking of voltage-gated calcium channels by cadmium did not prevent the Copine-6 response to glutamate (Supplementary Fig. 3c). Together, these data show that calcium influx via NMDA receptors triggers the translocation of Copine-6 into postsynaptic spines and its association to dendritic shaft membranes, and that the calcium transients triggered by an LTP-inducing paradigm are large enough to cause this fast and reversible translocation.

### Copine-6 is involved in hippocampal synaptic plasticity

To analyse the function of Copine-6 *in vivo*, we next generated Copine-6-deficient mice by breeding the lacZ knock-in mice (see Supplementary Fig. 1a) to homozygosity. The *Cpne6* KO mice were born at the expected Mendelian ratio and did not show any overt phenotype. In particular, body- or brain weight, or the overall morphology of the brain was not changed (Supplementary Fig. 4a). Moreover, except the loss of Copine-6, *Cpne6* KO mice did not show any difference in the levels of the postsynaptic scaffold proteins PSD95 and SynGAP, of AMPA and NMDA receptors subunits or the synapse modifiers CaMKII and Creb (Supplementary Fig. 4b). In addition, phosphorylation of neither CaMKII nor Creb was altered (Supplementary Fig. 4b). In addition, Copine-6 deficiency did not affect spine density and morphology in hippocampal pyramidal neurons (Supplementary Fig. 5a,b) as revealed by using transgenic Thy1-mGFP mice that express a membrane-targeted form of GFP sparsely in neurons of the hippocampus^31^. Consistent with the notion that synapses are not affected in their basal function and number, the frequency and amplitude of excitatory and inhibitory miniature postsynaptic currents were the same in wild-type (WT) and in *Cpne6* KO mice (Supplementary Fig. 5c,d).

**Figure 3:**
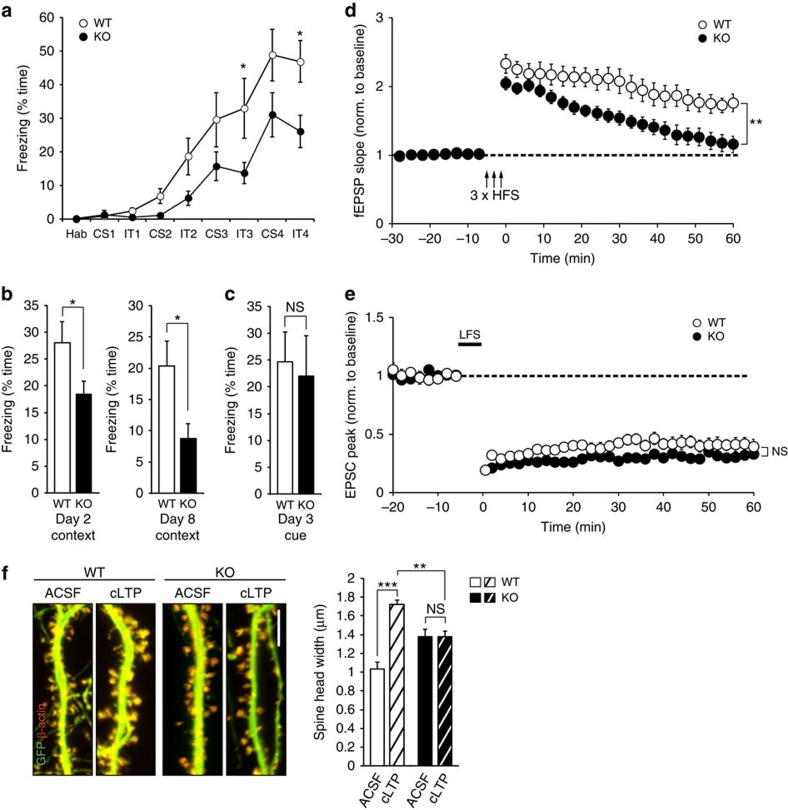
Copine-6 is required for hippocampal learning and memory and synaptic plasticity. (**a**–**c**) Fear conditioning in WT and *Cpne6* KO mice. (**a**) Freezing behaviour during training session on day 1. CS, conditioning stimulus (tone); Hab, habituation; IT, inter-training interval. *P<0.05 by two-way ANOVA with Bonferroni *post-hoc* test. (**b**) Time of freezing in context test on day 2 (left) or on day 8 (right). *P<0.05 by Student's *t*-test. (**c**) Freezing in cue test (cued fear memory) on day 3 after training. NS, nonsignificant. *P*>0.05 by Student's *t*-test. Data in **a**–**c** are mean±s.e.m. from *n*=9 WT and 10 KO male mice of 8 to 13 weeks of age. (**d**) Extracellular field potential recordings from acute slices. LTP was induced by high-frequency stimulation (HFS). Data are mean±s.e.m. from WT: *n*=9 recordings; KO: *n*=7 recordings (6 WT mice; 5 KO mice; age between 6 and 8 weeks). **P<0.01; by two-way ANOVA with Bonferroni *post-hoc* test. (**e**) Whole-cell patch-clamp recordings from CA1 neurons of acute slices. LTD was induced by low-frequency stimulation (LFS) and simultaneous postsynaptic depolarization. Data are mean±s.e.m. from WT: *n*=9 recordings; KO: *n*=10 recordings (7 WT mice, 5 KO mice; age between 4 and 5 weeks). NS. *P*>0.05 by two-way ANOVA with Bonferroni *post-hoc* test. (**f**) DIV21 cultured hippocampal neurons isolated from WT or *Cpne6* KO mice, which were transfected at DIV14 with constructs coding for β-actin-tdRFP (red) and GFP (green). Cultures were incubated with ACSF or cLTP-inducing ACSF (cLTP) for 10 min and spine head width was determined after 40 min. Data are mean±s.e.m. from WT ACSF, WT cLTP, KO cLTP: *n*=9 neurons; KO ACSF: *n*=11 neurons (from 3 independent cultures per genotype). ***P<0.001; **P<0.01; NS. *P*>0.05 by one-way ANOVA with Tukey's *post-hoc* test. Scale bar, 10 μm.

As our data from cultured neurons strongly indicated a role of Copine-6 in activity-regulated processes, *Cpne6* KO mice were next tested for hippocampal learning and memory in a fear-conditioning paradigm. *Cpne6* KO mice showed a significant deficit in learning (Fig. 3a) and in a context-dependent memory (Fig. 3b). The observed behavioural change was not based on altered anxiety or locomotor activity, or changes in foot-shock sensitivity between WT and *Cpne6* KO mice (Supplementary Fig. 6). Notably, the *Cpne6* KO mice did not show any deficit in amygdala-dependent cued memory (Fig. 3c).

Deficits in hippocampal learning and memory significantly correlate with deficits in LTP^32^. To test whether LTP is affected in *Cpne6* KO mice, we used acute hippocampal slices and measured field excitatory postsynaptic potentials (fEPSPs) in CA1 pyramidal neurons by stimulation of Schaffer collaterals. Using three trains of high-frequency stimulation, a robust LTP was induced in WT mice (Fig. 3d). In contrast, after the initial increase in the EPSPs, responses in *Cpne6* KO mice returned to baseline within 60 min (Fig. 3d). Interestingly, NMDA receptor-dependent LTD, triggered by low-frequency stimulation at 1 Hz^19^, was not affected in *Cpne6* KO mice (Fig. 3e). The LTP deficit in *Cpne6* KO mice was not based on alterations of the input-output ratio of Schaffer collateral-CA1 connections (Supplementary Fig. 5e) or changes in the paired-pulse ratio (Supplementary Fig. 5f). In summary, these experiments show that Copine-6 is necessary for the long-lasting, stable strengthening of synapses in response to increased synaptic activity and for learning and memory.

It is well established that successful induction of LTP results in changes in spine structure, a process that is known as spine structural plasticity^2^,^33^. For example, activation of a single spine by glutamate-uncaging induces the enlargement and the synaptic strengthening of the stimulated spine^8^. The enlargement and strengthening of synapses is also observed on glycine stimulation, a procedure that induces cLTP^29^. To test whether Copine-6 is involved in spine structural plasticity, cultured hippocampal neurons were co-transfected with cytosolic GFP and β-actin-tdRFP (to label spines) at DIV14 and submitted to cLTP at DIV21. The size of the spine heads was measured 40 min after stimulation. Spine head width in neurons isolated from WT mice significantly increased (Fig. 3f), whereas it did not change in neurons isolated from *Cpne6* KO mice (Fig. 3f). We also noticed that non-stimulated spines in the *Cpne6* KO neurons were larger than in WT but their size was still significantly smaller than WT spines after cLTP. These results show that Copine-6 is essential for spine structural plasticity and they indicate that the failure to increase spine size is the structural basis for the observed deficiency in LTP, learning and memory in *Cpne6* KO mice.

### A calcium mutant of Copine-6 inhibits structural plasticity

**Figure 4:**
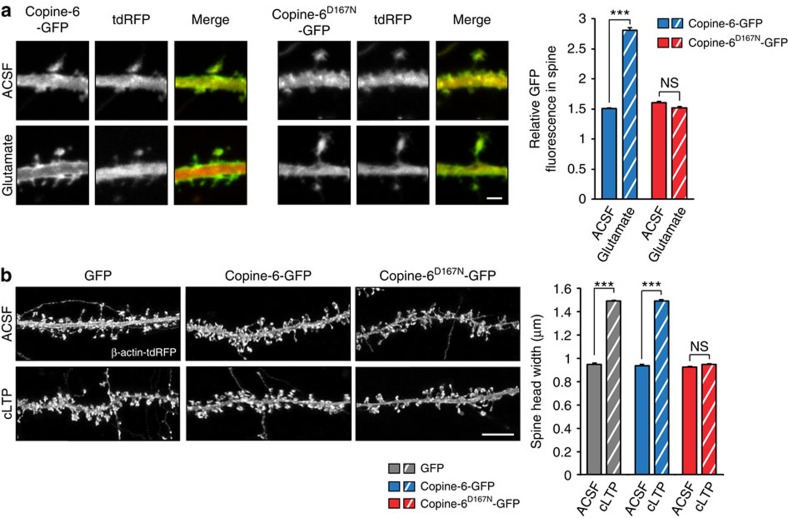
The calcium Copine-6^D167N^ mutant does not translocate into spines and inhibits spine structural plasticity. (**a**) DIV14 hippocampal cultures, which had been co-transfected at DIV7 with constructs encoding Copine-6-GFP or Copine-6^D167N^-GFP together with tdRFP were incubated for 5 min in ACSF or ACSF supplemented with 100 μM glutamate and 10 μM glycine (glutamate). Right: quantification of the relative GFP intensity in spines after normalization to tdRFP. Data are mean±s.e.m. from *n*=500 spines per condition (25 spines per neuron from 20 neurons of each condition; from 3 independent cultures). ***P<0.001; NS, nonsignificant. *P*>0.05 by one-way ANOVA with Tukey's *post-hoc* test. Scale bar, 2 μm. (**b**) DIV21 hippocampal cultures, which had been transfected at DIV14 with constructs encoding Copine-6-GFP or Copine-6^D167N^-GFP together with β-actin-tdRFP. Cultures were incubated with ACSF or cLTP-inducing ACSF (cLTP) for 10 min and analysed after additional 40 min. Left: representative pictures of β-actin-tdRFP fluorescence and GFP fluorescence. Right: quantification of spine head width. It is noteworthy that expression of Copine-6^D167N^-GFP abrogates the increase in spine head width seen in GFP- and Copne-6-GFP-expressing neurons on induction of cLTP. Data are mean±s.e.m. from *n*=1,000 spines per condition (50 spines per neuron from 20 neurons of each condition; from 3 independent cultures). ***P<0.001; NS. *P*>0.05 by one-way ANOVA with Tukey's *post-hoc* test. Scale bar, 10 μm.

To address the role of calcium binding for the function of Copine-6, we next generated a mutant that does not respond to calcium. To do this, we mutated the aspartate residue at position 167 of Copine-6 to asparagine (Copine-6^D167N^; Supplementary Fig. 7a) because of its high similarity to the calcium-binding motif in other C2 domain-containing proteins, such as protein kinase C or synaptotagmin^34^,^35^. In Copine-6, this mutation abolished ionomycin-induced aggregation of Copine-6 at plasma membranes in transfected COS7 cells (Supplementary Fig. 7b) and cell fractionation experiments showed that the mutated protein remained in the cytosol in the presence of calcium (Supplementary Fig. 7c). To test whether the mutant would also not respond to calcium influx in neurons, we next compared the dynamics of the GFP fluorescence in DIV14 cultured hippocampal neurons that were transfected at DIV7 to express either Copine-6-GFP or Copine-6^D167N^-GFP together with tdRFP (as a volume marker). Glutamate stimulation of neurons for 5 min, which leads to a strong calcium influx via synaptic and non-synaptic glutamate receptors^36^, did not result in the accumulation of Copine-6^D167N^-GFP in spines, while Copine-6-GFP accumulated strongly (Fig. 4a). These results show that calcium binding of Copine-6 requires the aspartate residue at position 167, and that the calcium binding is essential for the enrichment of Copine-6 in postsynaptic spines after stimulation. To test whether overexpression of Copine-6^D167N^ would affect spine structural plasticity, we analysed cLTP-induced spine structural plasticity in neurons that were double transfected with β-actin-tdRFP (to identify spines) and with constructs encoding GFP (as a control), Copine-6-GFP or Copine-6^D167N^-GFP. Although cLTP increased the spine head width in GFP- and Copine-6-GFP-expressing neurons, expression of Copine-6^D167N^-GFP prevented spine structural plasticity (Fig. 4b). Thus, neurons expressing the calcium-insensitive mutant of Copine-6 do not respond to cLTP, as do *Cpne6* KO neurons (Fig. 3f), demonstrating that Copine-6^D167N^ acts as a dominant-negative mutant in this paradigm. Transfection of either construct did not significantly affect the number of spines (Supplementary Fig. 7d). Thus, these results support the notion that Copine-6 is critically involved in spine structural plasticity, and that this function requires the translocation of Copine-6 into postsynaptic spines on the increase in intracellular calcium.

### Copine-6 binds Rac1 and affects its activity

**Figure 5:**
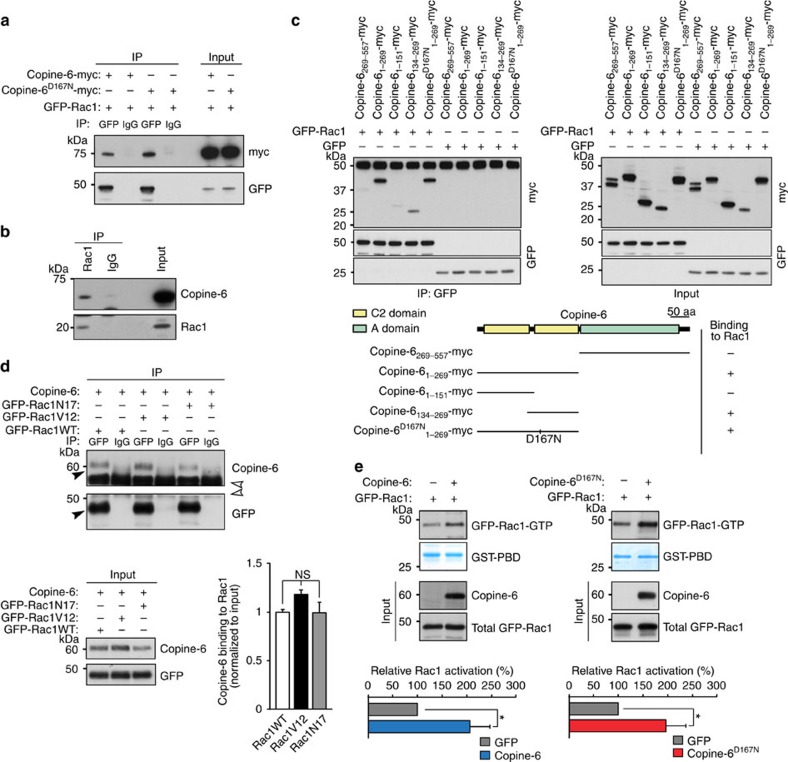
Characterization of Copine-6 binding to Rac1 and regulation of Rac1 activity. (**a**) COS7 cells were co-transfected with expression constructs encoding GFP-Rac1 and Copine-6-myc or Copine-6^D167N^-myc. Rac1 was immunoprecipitated (IP) using anti-GFP antibodies (GFP) or non-immune IgG (IgG) as control. Immunoprecipitates were analysed with anti-myc (myc) or anti-GFP antibodies. (**b**) Hippocampal lysates of 6-week-old mice after immunoprecipitation (IP) with anti-Rac1 antibodies or non-immune IgG. Precipitates were analysed with anti-Copine-6 (Copine-6) or anti-Rac1 (Rac1) antibodies. Input represents western blottings of hippocampal lysates. (**c**) COS7 cells were co-transfected with expression constructs encoding myc-tagged Copine-6 deletion mutants together with GFP-Rac1 or GFP (as negative control). Left: analysis of anti-GFP immunoprecipitates (IP) using antibodies to myc or GFP. Right: western blot analysis of COS7 cell lysates before immunoprecipitation. Bottom: schematic illustration of deletion constructs and summary of results. (**d**) COS7 cells were co-transfected with Copine-6 and GFP-Rac1 (GFP-Rac1WT), a dominant-negative form of Rac1 (GFP-Rac1N17) or a constitutively active form (GFP-Rac1V12). Immunoprecipitations (top) were performed using anti-GFP antibodies (GFP) or non-immune IgG (IgG) as a control. Immunoprecipitates were analysed with antibodies to Copine-6 or GFP as indicated. Black arrowheads indicate Copine-6 and GFP-Rac1, respectively; open arrowheads indicate IgG heavy chains. Bottom: western blot analysis of COS7 cell lysate (left; input) and quantification of the relative binding of Copine-6 to the different Rac1 mutants (right). Data are mean±s.e.m. from *n*=3 experiments. NS, nonsignificant. *P*>0.05 by one-way ANOVA with Tukey's *post-hoc* test. (**e**) Determination of the relative amount of activated Rac1 (Rac1-GTP) using GST-PBD-mediated precipitation from COS7 cells co-expressing GFP-Rac1 together with empty vector (as negative control), Copine-6 (left) or Copine-6^D167N^ (right). Western blotting of cell lysates before GST-PBD precipitation (input) is shown and was used for normalization of the quantification (bottom). Data are mean±s.e.m. from Copine-6: *n*=4 cultures; Copine-6D167N: *n*=5 cultures. *P<0.05 by Student's *t*-test.

Several lines of evidence indicate that spine structural plasticity involves changes in the actin cytoskeleton^8^,^37^,^38^. As our results show that Copine-6 is required for spine structural plasticity, we searched for possibilities of how Copine-6 could affect actin cytoskeleton dynamics. Actin dynamics is regulated by Rho-like GTPases and they have also been implicated in neuronal activity-induced changes of synapse structure^39^,^40^. In co-transfection experiments in COS7 cells, myc-tagged versions of Copine-6 or Copine-6^D167N^ co-immunoprecipitated with GFP-Rac1 (Fig. 5a). Importantly, the interaction between Copine-6 and Rac1 was also detected in lysates from hippocampi (Fig. 5b) and the total levels of Rac1 were not altered in *Cpne6* KO hippocampi (Supplementary Fig. 4b). To determine the interaction domain between Rac1 and Copine-6, deletion constructs of Copine-6 were co-expressed with GFP-Rac1 and lysates were immunoprecipitated with antibodies to GFP. These mapping studies showed that the second C2 domain of Copine-6 was both required and sufficient for Rac1 binding (Fig. 5c). Interestingly, mutation of the calcium-binding aspartate at position 167 of Copine-6 did not affect its binding to Rac1 (Fig. 5c). In addition, the GTPase activity of Rac1, tested by using a constitutively active (Rac1V12) or a dominant-negative form (Rac1N17), did not affect its interaction with Copine-6 (Fig. 5d). As Copine-6 does not share any sequence similarity to known GTPase-activating proteins (GAPs) or guanine nucleotide exchange factors (GEFs), we tested whether Copine-6 binding would affect Rac1 activity indirectly. To this end, Copine-6 and Rac1 were co-expressed in COS7 cells and the amount of activated Rac1-GTP was determined. Compared with the transfection with empty vector, co-expression of Copine-6 or Copine-6^D167N^ resulted in a strong increase in the total amount of active Rac1-GTP (Fig. 5e). Thus, these results show that (1) Copine-6 interacts with the active and the inactive form of Rac1, (2) Copine-6 boosts Rac1 activation, and (3) binding and activation of Rac1 by Copine-6 is independent of its calcium binding.

**Figure 6:**
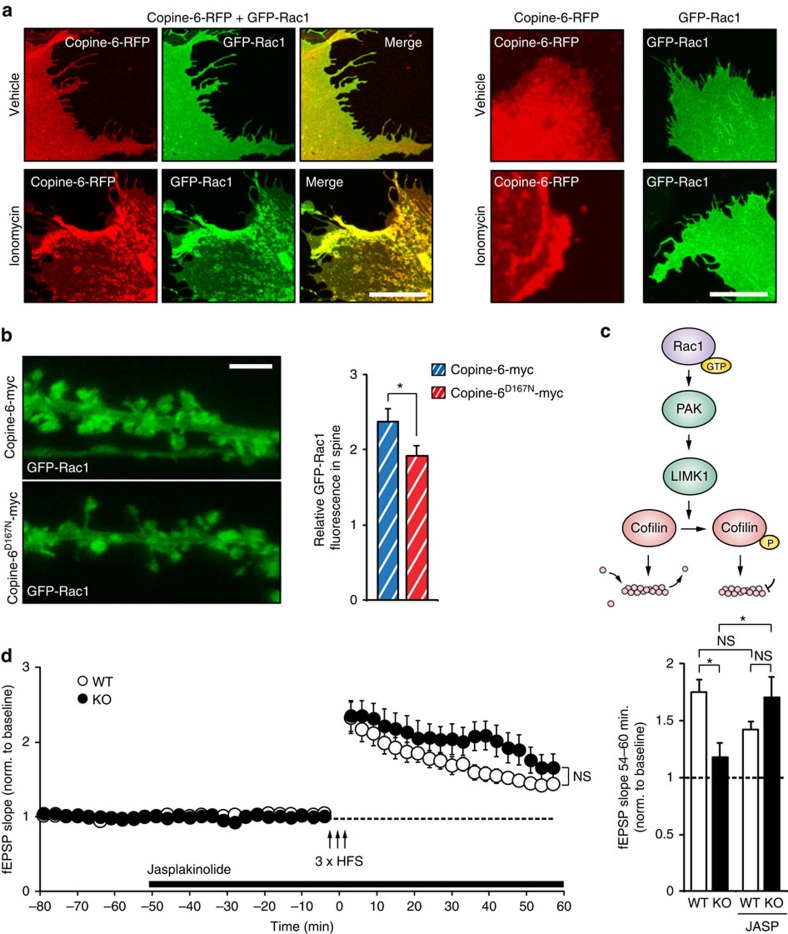
Copine-6 regulates the dynamics of the actin cytoskeleton. (**a**) COS7 cells expressing the indicated proteins were treated with vehicle or with the calcium ionophore ionomycin. Co-expression of Copine-6-RFP with GFP-Rac1 (left) resulted in the localization of GFP-Rac1 to membranes on ionomycin treatment. In COS7 cells expressing only GFP-Rac1 (right), ionomycin did not affect the cellular localization of GFP-Rac1. Scale bar, 20 μm. (**b**) DIV21 hippocampal cultures, which had been transfected at DIV19 with constructs encoding Copine-6 or Copine-6^D167N^ together with GFP-Rac1 and tdRFP. Cultures were incubated with cLTP-inducing ACSF for 10 min. Right: quantification of the relative GFP-Rac1 intensity in spines after normalization to tdRFP. Data are mean±s.e.m. from *n*=36 spines from 6 neurons per conditions. *P<0.05 by Student's *t*-test. Scale bar, 3 μm. (**c**) Scheme of the Rac1-PAK-LIMK1-Cofilin pathway. (**d**) Extracellular field potential recordings from acute slices of 6- to 8-week-old WT or *Cpne6* KO mice. Slices were treated with jasplakinolide during recordings as indicated. LTP was induced by high-frequency stimulation (HFS). Data are mean±s.e.m. from *n*=7 slices and mice per genotype. NS, nonsignificant. *P*>0.05 by two-way ANOVA with Bonferroni *post-hoc* test. For quantification (right), the mean of the normalized fEPSP slope 54–60 min after HFS was compared. Data are mean±s.e.m. from *n*=7–9 recordings per condition (from 6 to 7 mice per genotype, age between 6 and 8 weeks). *P<0.05; NS, nonsignificant. *P*>0.05 by one-way ANOVA with Tukey's *post-hoc* test.

As spine structural plasticity in cultured hippocampal neurons was prevented in neurons that express the Copine-6^D167N^ mutant, we next asked as to how calcium would affect the interaction of Copine-6 with Rac1. To approach this, COS7 cells were co-transfected with Copine-6-RFP and GFP-Rac1, and calcium influx was triggered by ionomycin. In cells expressing both constructs, ionomycin induced the clustering of Rac1 at membranes and those clusters were co-localized with Copine-6 (Fig. 6a). In cells expressing only GFP-Rac1, treatment with ionomycin did not cause any clustering of Rac1 to membranes (Fig. 6a). The co-localization of Rac1-GFP and Copine-6 was also seen in cultured hippocampal neurons after stimulation with glutamate (Supplementary Fig. 8a). Most importantly, Rac1 localization in neurons was affected by the calcium mutant of Copine-6 as demonstrated by the finding that Rac1-GFP did not accumulate in spines of on cLTP induction in the presence of the calcium mutant Copine-6^D167N^-myc, whereas this was the case in control neurons expressing Copine-6-myc (Fig. 6b). These experiments support the notion that Copine-6 is involved in the recruitment of Rac1 into spines. In the presence of the calcium mutant Copine-6^D167N^, which does not translocate to spines after cLTP induction but still binds to Rac1, this recruitment is prevented. Experiments in COS7 cells showed that Copine-6 did not affect the localization of RhoA or Cdc42 on ionomycin treatment (Supplementary Fig. 8b), demonstrating that association of Copine-6 is specific for Rac1.

Rac1 activation is known to cause phosphorylation of p21-activated kinases (PAKs)^41^, which in turn activate LIMK1 to alter phosphorylation of Cofilin (Fig. 6c)^42^. By transfecting COS7 cells with Copine-6, Rac1 and PAK1, we investigated co-clustering after ionomycin treatment. Indeed, plasma membrane-associated clusters of Copine-6 and Rac1 were also enriched for PAK1 (Supplementary Fig. 8c, top and bottom panels). Interestingly, the PAK1 clusters were also positive for its phosphorylated form (p-PAK1), consistent with the experiments that Copine-6 potentiates Rac1 activation. The clustering of both Rac1 and PAK1 at plasma membranes was not observed when RFP instead of Copine-6 was transfected into COS7 cells (Supplementary Fig. 8c, middle panel). Thus, these results are evidence that calcium binding of Copine-6 recruits the Rac1–PAK complex to plasma membranes, and that Rac1 is activated. In analogy, local calcium signals triggered by LTP induction may thus recruit this protein complex to spines and result in the re-arrangement of the cytoskeleton. As the calcium-binding mutant of Copine-6 does not respond to calcium, Rac1 and PAK1 will not be recruited to spines, the actin cytoskeleton will not be remodelled and thus the size of spine heads will not increase.

To test whether the entire Rac1-PAK-LIMK1-Cofilin pathway was affected by Copine-6, we also measured LTP-induced enrichment of Cofilin in spines, which precedes and is required for spine structural plasticity^43^. In contrast to WT neurons, Cofilin was not significantly increased in spines of *Cpne6* KO neurons on cLTP (Supplementary Fig. 9a), indicating defective Rac1-PAK-LIMK1-Cofilin signalling in spines. In addition, phosphorylation of Cofilin was decreased in *Cpne6* KO hippocampal cultures compared with WT neurons (Supplementary Fig. 9b). As this pathway controls actin dynamics, we finally tested the effect of jasplakinolide, a toxin that stabilizes newly formed actin filaments^44^, and has been shown to restore LTP under conditions where actin stabilization is weakened^39^,^45^. Addition of jasplakinolide during field recordings to acute hippocampal slices from *Cpne6* KO mice resulted in the robust induction of LTP, which was no longer different from slices isolated from WT mice (Fig. 6d). Thus, short-term treatment of KO slices with the actin stabilizer jasplakinolide is sufficient to fully restore the LTP deficit of Copine-6-deficient CA1 neurons. Together, these data strongly support that translocation of Copine-6 by LTP-inducing stimulation results in local activation of Rac1 in spines and subsequent stabilization of the subsynaptic actin cytoskeleton.

## Discussion

There is strong evidence that the increase in spine size correlates with the strengthening of synapses as a result of LTP-inducing paradigms^46^. Learning paradigms in the motor and the sensory system have been shown to cause the formation of long-lasting spines^47^,^48^, indicating that changes in spine structure are the cellular correlates of memory. As the structural changes are synapse specific^8^, spatial restriction of the signal is an important aspect in this process. Moreover, it is well established that changes in the synaptic actin cytoskeleton are necessary for translating the local, presynaptic activity into structural changes of the postsynaptic spines.

Here we characterize Copine-6 and show that it affects spine structural plasticity in response to presynaptic activity. Specifically, we found that Copine-6 becomes localized to postsynaptic spines by experimental paradigms that mimic local calcium influx into postsynaptic spines (Fig. 2). The recruitment of Copine-6 to spines was not observed in the calcium mutant Copine-6^D167N^ (Fig. 4a) and, most importantly, *Cpne6* KO neurons or neurons overexpressing the Copine-6^D167N^ mutant did not respond with an increase in the size of postsynaptic spine heads when subjected to cLTP (Figs 3f and 4b). The absence of structural synaptic plasticity in the *Cpne6* KO mice correlated with the lack of LTP maintenance (Fig. 3d) and the impairment of hippocampus-dependent learning and memory (Fig. 3a–c). Interestingly, the initial increase in the postsynaptic current after tetanic stimulation of the afferents was not affected in the *Cpne6* KO mice but this initial potentiation was not maintained after 60 min, further corroborating Copine-6's role in spine structural plasticity (Fig. 3d).

In addition, here we provide evidence that Copine-6's role in spine structural plasticity is mediated by its binding and regulatory function towards Rac1 (Fig. 5). Importantly, the response of Copine-6 to be recruited to plasma membranes and to postsynaptic spines, which is a consequence of an increase in intracellular calcium, causes co-recruitment of Rac1 (Fig. 6a). The recruitment of Rac1 to postsynaptic membranes and its activation on a short, 3-min incubation with NMDA has also been described in mouse hippocampus^49^. This recruitment and activation of Rac1 can be blocked by the NMDA receptor antagonist AP5, suggesting that it is triggered by NMDA receptor-mediated calcium influx^49^. Our data suggest that it might be Copine-6 that is responsible for the recruitment and local activation of Rac1. This, in turn, would then activate the Rac1-PAK-LIMK1-Cofilin pathway and cause actin re-arrangement. Indeed, cLTP induction resulted in the enrichment of Cofilin in WT spines and this enrichment required Copine-6 as seen in *Cpne6* KO neurons (Supplementary Fig. 9a). Moreover, jasplakinolide recovered LTP in *Cpne6* KO mice (Fig. 6d), further suggesting that Copine-6 indeed acts via the actin cytoskeleton.

Rac1 associates with Copine-6 in the inactive and the GTP-bound (that is, active) form (Fig. 5d), and this complex also includes the phosphorylated form of PAK1 (Supplementary Fig. 8b). The activity of Rho-GTPases is controlled by GAPs or GEFs but Copine-6 does not encode any domains reminiscent of such function. Thus, Rac1 recruited by Copine-6 to spines is likely to be activated by PSD-residing enzymes. Several GEFs and GAPs are indeed enriched in the PSD of glutamatergic synapses^50^,^51^ and some of these candidate RhoGEFs such as kalirin-7, Tiam1 or β-PIX have also been shown to be activated by increased neural activity^52^,^53^,^54^. Finally, changes in GEF activity affect spine morphology^53^,^55^,^56^ and their dysregulation has been implicated in synaptic pathologies such as mental retardation and schizophrenia^57^. Our results therefore indicate that the translocation of Copine-6 to postsynaptic spines generates a scaffold that brings Rac1 into close proximity to the RhoGEFs or RhoGAPs that are anchored to the postsynaptic scaffold. This will then allow Rac1 to locally activate the PAK-LIMK1-Cofilin pathway and to locally modulate actin cytoskeletal dynamics. In this context, it is interesting to note that the concentration of Copine-6 (Fig. 2) and Cofilin^43^ increases in spines when cLTP is triggered in neurons. In contrast, cLTP increases only the total amount but not the concentration of other postsynaptic scaffold molecules such as PSD-95, Homer1b and Shank1b^43^. These data indicate that Copine-6 and Cofilin belong to the same subgroup of postsynaptic proteins that are actively enriched in spines by LTP and thus corroborate our data that there is a functional relationship between Copine-6 and the Rac1-PAK-LIMK1-Cofilin pathway.

The best-characterized molecular mechanism that strengthens excitatory synapses after NMDA receptor activation involves CaMKII, a protein that affects many pathways involved in synaptic function^10^. Genetic depletion or acute inhibition of CaMKIIα prevents initial synaptic strengthening on LTP induction^58^,^59^ by inhibiting phosphorylation of NMDA receptors^10^. Besides this early process, CaMKII also affects LTP maintenance through, for example, regulation of the actin cytoskeleton and spine structural plasticity. However, the molecular details underlying these structural activities of CaMKII are less well understood, mainly because of the difficulty to develop agents that allow selective interference in LTP maintenance without disturbance of early LTP. It is also interesting to note that pharmacological inhibition of CaMKII does not fully abolish the increase in spine volume 30 min after glutamate uncaging^60^, suggesting that other pathways contribute to the spine enlargement during LTP. One of those contributors could be the here-described Copine-6-Rac1-PAK-LIMK1-Cofilin pathway.

The mechanism of LTP maintenance is thought to be largely based on increased incorporation of AMPA receptors into the postsynaptic membrane, a process that involves changes in the dynamics of the actin cytoskeleton and correlates with an increase in the size of the postsynaptic spines^8^. Interestingly, site-directed mutagenesis in *C. elegans* has identified the Copine *NRA-1* as an essential component for synaptic levels of the levamisole receptor (orthologue of the nicotinic acetylcholine receptor)^16^.

Copine-6 expression in mice is reminiscent of proteins involved in synapse formation, maintenance and plasticity as it starts postnatally and stays high in the adult brain. In mice, *Cpne6* expression is strongest in the hippocampus with only little expression in the cerebral cortex (Fig. 1). In the human brain, *Cpne6* expression is also high in the hippocampus but has also been detected in the prefrontal cortex^23^,^61^. Interestingly, the peak of *Cpne6* expression in the human prefrontal cortex is highest at 5 years of age and is conspicuously shifted compared with non-human primates, such as chimpanzees and rhesus macaques, where *Cpne6* expression peaks at the age of <1 year^61^,^62^. The same temporal shift has been noted for several other synaptic genes (for example, *CAMK2*, *BDNF* and *NRXN3*) that are well known for their role in synaptic function^61^,^62^. This observation has led to the hypothesis that the temporal shift in the peak expression of those synaptic genes could contribute to the superior cognitive function of the human prefrontal cortex compared with non-human primates^62^. Thus, our discovery that Copine-6 indeed plays an important role in synaptic plasticity is further support for this hypothesis.

Finally, our description of the role of Copine-6 in the mouse brain may foster the functional analysis of other members of the Copine family, as they are structurally highly similar but are expressed in distinct brain regions and at different time points in development^22^,^23^. Moreover, Copines have different affinities for calcium and translocate to membranes with different rates^15^. Thus, Copines may respond to different patterns of neuronal activity. Finally, the Rac1-PAK-LIMK1-Cofilin pathway has been implicated in several diseases that affect memory including Fragile X syndrome, Alzheimer's disease and Williams–Beuren syndrome^57^, and the discovery that Copine-6 regulates this pathway may provide new avenues for the better understanding of the molecular mechanisms involved in those pathologies.

## Methods

### Mice

*Cpne6* KO mice were generated by homologous recombination (illustrated in Supplementary Fig. 1a), using a cassette in which the entire protein coding region of *Cpne6* (exon 2–16) was replaced by nls-LacZ. Gene targeting was carried out in embryonic stem cells (75% C57BL/6, 25% 129 Sv). The targeting vector contained 5 kb of 5′-arm (*Cpne6* promoter, exon 1, intron 1 and a small part of exon 2) followed by nls-LacZ-SV40 polyA signal-LoxP-PGKneo-LoxP and 3 kb of 3′-arm (*Cpne6* exon 16, intron 16, exon 17 and 3′-downstream sequence) followed by MC1-HSV-TK (for negative selection). Chimeric mice were obtained by microinjection of correctly targeted embryonic stem cell clones into C57BL/6 blastocysts. The LoxP-flanked neo-cassette was removed by intercrossing mice with *Hprt-cre* (Cre-deleter) mice. Genotypes were identified by PCR flanking the 5′-end with following specific primers: Gt5F (5′-TGC AGC ACT GGC TCA TAG AC-3′), Gt5R (5′-GGA AAG TCC TTG GGG TCT TC-3′) and Gt5wtR (5′-GCA CCC ATC CCA TCT CTG-3′) or at the 3′-end: Gt3R (5′-ATC TGA GGC ATG ACG GGT AG-3′), Gt3wtF (5′-CTA TGA CTC CCA GCC CTA GC-3′) and Gt3F (5′-CAC TGC ATT CTA GTT GTG GTT TG-3′). Mice, except those for behavioural studies, were on a mixed C57BL/6;129 Sv background and *Cpne6* KO and WT mice derived from the same litter. All mouse experiments were performed according to the federal guidelines for animal experimentation and approved by the authorities of the Canton Basel-Stadt.

### Behavioural studies

Behavioural experiments were performed with a cohort of male mice that were backcrossed to C57BL/6 mice for at least ten generations. In the open-field test, explorative behaviour and locomotor activity was monitored by video tracking for 10 min in a circular open field. Foot-shock sensitivity was measured in an independent group of mice. After 2 min habituation, foot shocks with a duration of 2 s were administered every 30 s, starting at 0.05 mA with a 0.05-mA increment between each shock. Flinching, jumping and vocalization were scored blinded to genotype. For fear-conditioning experiments, mice were trained on day 1 as follows: mice were allowed to habituate to the test chamber for 2 min before the onset of four consecutive training blocks. Each training block consisted of a 30s tone stimulus (5,000 Hz, 80 dB) co-terminated with 2 s foot shock (0.7 mA) followed by 2 min inter-trial interval. On day 2, contextual fear memory was assessed by placing the mice in the training chamber for 12 min. On day 3, cued fear memory was tested in a novel chamber, which was distinct from the training chamber. After 2 min habituation, tone stimulus was presented and freezing behaviour was monitored for 3 min. On day 8, contextual fear memory in the training box was assessed in the same way as on day 2. Most of the mice used for fear-conditioning experiments were earlier used for open-field tests.

### DNA constructs

The complementary DNAs (cDNAs) encoding Copine-6 and β-actin were cloned from reverse-transcribed mRNA isolated from rat brain with following primers: Copine-6: ss HindIII 5′-CCC AAG CTT AGT GCC ATG TCG GAC CCA GAG ATG GGA TGG GTG CCT GAG C-3′ and as BamHI 5′-CGC GGA TCC TGG GCT GGG GCT GGG-3′ for fusion to GFP or including STOP codon: as BamHI 5′-CGC GGA TCC TCA TGG GCT GGG GCT GGG-3′, β-actin: ss EcoRI 5′-CCGGAATTCTTCGCCATGGATGAC-3′ and as BamHI 5′-CGC GGA TCC GAA GCA TTT GCG GTG CAC-3′. For expression in COS7 cells, cDNAs were subcloned into pEGFP(N3) (BD Bioscience, Clontech), pEGFP(C1) (BD Bioscience, Clontech) and pIRES2-EGFP (BD Bioscience, Clontech). For expression in cultured hippocampal neurons, cDNAs were subcloned into pMH4-SYN-1 (gift from T. G. Oertner; Friedrich Miescher Institute for Biomedical Research, Basel). tdRFP fusion constructs were generated by replacing enhanced GFP (EGFP) with tdRFP sequence as previously described^63^. pEGFP-Rac1, pEGFP-Cdc42 and pEGFP-RhoA were a gift from A. W. Püschel (University of Münster, Germany). Copine-6^D167N^ was generated from cDNA encoding Copine-6 as a template by single primer mutagenesis using primer: 5′-CAC AAG CTG GAT AAC AAG AAT CTG TTC AGC AAG TCT G-3′. Rac1V12 was generated from a Rac1 template by single primer mutagenesis using primer 5′-AGA CGT AAG CTG TTG GTA AAA CCT-3′ and Rac1N17 using primer 5′-AGA CGG AGC TGT TGG TAA AAA CT-3′.

### Antibodies

For immunostaining, co-immunoprecipitation and western blot analysis, the following antibodies were used: β-actin (Cell Signaling, Cat. 4970; 1:1,000), β-gal (Abcam, Cat. ab9361; 1:200), β-tubulin (BD Pharmigen, Cat. 556321; 1:5,000), Copine-6 (clone 42, Santa Cruz, Cat. sc-136357; 1:1,000 for western blot analysis and 1:200 for immunostaining), CaMKII (Chemicon, Cat. MAB8699; 1:1,000 for western blot analysis and 1:200 for immunostaining), p-CaMKII (Cell Signaling, Cat. 3361; 1:1,000), Cofilin (Abcam, Cat. ab11062; 1:1,000), Creb (Cell Signaling, Cat. 9197; 1:1,000); p-Creb (Cell Signaling, Cat. 9198; 1:1,000), Flotillin-1 (BD Bioscience, Cat. 610820; 1:1,000), GAD67 (Chemicon, Cat. MAB5406; 1:1,000), GFAP (Millipore, Cat. MAB360; 1:1,000), GFP (Roche, Cat. 11814460001; 2 μg for co-immunoprecipitation), GluR2 (BD Pharmigen, Cat. 556341; 1:5,000), myc (clone 9E10, produced and purified from hybridoma cell line 9E10; 2 μg for co-immunoprecipitation), Na^+^/K^+^-ATPase (GeneTex, Cat. GTX22872; 1:1,000), NeuN (Millipore, Cat. MAB377; 1:1,000), NR1 (BD Pharmigen, Cat. 556308; 1:1,000), NR2A (Millipore, Cat. 07-632; 1:1,000), NR2B (Millipore, Cat. 06-600; 1:1,000), p-PAK (Life Technologies, Cat. 44940G; 1:150), PSD95 (Affinity Bioreagents, Cat. MA1045; 1:1,000), Rac1 (Cell Signaling, Cat. 2465 for western blot analysis and 1:1,000, Millipore, Cat. 05-389, 2 μg for co-immunoprecipitation) and SynGAP (Affinity Bioreagents, Cat. PA1046; 1:1,000).

### Quantitative real-time PCR

Quantitative real-time PCR was performed on cDNA samples made from RNA collected from hippocampal cultures using SYBR Green PCR Core Reagents (Applied Biosystems) on an ABI 7000 and 7700 Sequence Detection System (Applied Biosystems). The following primers were used: Copine-6s: (5′-CCC CAA GTA CCG AGA CAA GAA GA-3′), Copine-6as (5′-GGA GGC TGT GAA GTC GAT AGC-3′), GAPDHs (5′-CATC GTG GAA GGG CTC ATG AC-3′) and GAPDHas (5′-CTT GGC AGC ACC AGT GGA TG-3′).

### Tissue preparation from mouse brains

Hippocampi were dissected on ice and homogenized in lysis buffer (50 mM Tris-HCl pH 7.5, 5 mM EDTA, 150 mM NaCl, 1% NP-40 and 0.5% sodium deoxycholate, including proteases and phosphatase inhibitor cocktails (Roche)) by a glass/Teflon homogenizer. Insoluble material was removed by centrifugation (16,000 *g*, 15 min, 4 °C). For western blot analysis, the protein concentration was determined by BCA assay (Pierce) and samples were boiled in SDS–PAGE loading buffer for 5 min at 95 °C. Equal amounts of total protein were loaded on SDS–PAGE.

### Histochemistry and immunohistochemistry

Mice were transcardially perfused with 4% paraformaldehyde in PBS (4% PFA/PBS) and dissected tissue was postfixed overnight in 4% PFA/PBS. For β-galactosidase staining, brains were cryoprotected (30% sucrose, overnight) and embedded in O.C.T. Sections (100-μm-thick) were cut on a cryostat. Free-floating sections were washed in PBS and then incubated three times for 30 min in rinse buffer (100 mM sodium phosphate buffer pH 7.3, 2 mM MgCl_2_, 0.01% sodium deoxycholate and 0.02% NP-40). For staining, sections were incubated overnight at 37 °C in the dark in staining solution (5 mM potassium ferricyanide, 5 mM potassium ferrocyanide and 1 mg ml^−1^ X-gal). Stained sections were postfixed for 2 h at 4 °C in 10% formalin and mounted on glass slides.

For immunohistochemistry, brains were embedded in paraffin and 5-μm-thick sections were cut on a microtome. Sections were deparaffinized with xylene and rehydrated by serial ethanol steps. Antigen retrieval was performed in boiling citrate buffer (10 mM sodium citrate pH 6 and 0.05% Tween-20) for 20 min. After washing in PBS, sections were incubated for 30 min at room temperature (RT) in blocking solution (5% BSA and 0.2% Triton X-100 in PBS). Subsequently, sections were incubated with primary antibody in blocking solution overnight at 4 °C. After extensive washing with PBS, sections were incubated for 1 h at RT with the appropriate secondary antibody and 4,6-diamidino-2-phenylindole. Coverslips were mounted with Celvol (Celanese).

### COS7 cell culture

COS7 cells were maintained in DMEM medium (Invitrogen) in the presence of 10% fetal bovine serum (FBS; Gibco). Transfections were performed with Lipofectamin 2000 (Invitrogen) following the manufacturer's instructions; cells were analysed 24 h after transfection. For ionomycin treatment, cells were washed twice with 37 °C PBS and incubated for 4 min at 37 °C in 2 μM ionomycin (Sigma-Aldrich, Cat. I9657) in DMEM. For cholesterol depletion, COS7 cells were incubated for 1 h at 37 °C with 10 mM methyl-β-cyclodextrin (Sigma-Aldrich, Cat. C4555) in complete medium. Cells were fixed with 4% PFA/PBS for 20 min. Lipid raft staining was performed with 1 μg ml^−1^ Choleratoxin subunit B1 (Molecular Probes; C-34778 conjugated with Alexa 647) for 1 h at RT.

Rac1 activation was measured in COS7 cells that were depleted of FBS for 4 h. Cells were washed twice with ice-cold PBS and lysed in ice-cold Mg^2+^ lysis/wash buffer (Millipore, Cat. 20–168). Lysates were precleared with glutathione Sepharose beads and insoluble material was removed by centrifugation (14,000 *g*, 1 min, 4 °C). Cleared lysates were incubated with 5 μg GST-PBD substrate (Millipore, Cat. 14–325) for 1 h at 4 °C followed by extensive washing with Mg^2+^ lysis/wash buffer. Proteins bound to beads were extracted by addition of SDS–PAGE loading buffer and boiling for 5 min at 95 °C. After centrifugation, supernatant was loaded on SDS–PAGE.

### Subcellular fractionation of COS7 cells

Fractionation was performed as described previously^64^. In brief, COS7 cells were washed twice with ice-cold PBS and scraped in ice-cold hypotonic lysis buffer (10 mM HEPES pH 7.5, 10 mM NaCl, 1 mM KH_2_PO_4_, 5 mM NaHCO_3_, 0.5 mM MgCl_2_ and protease inhibitor cocktail (Roche)) in the presence or absence of either 1 mM CaCl_2_, 10 nM EGTA or 2 mM EDTA. After homogenization by a glass/Teflon homogenizer, sucrose was added to a final concentration of 227 mM and carefully mixed. Nuclei and cell debris were removed by centrifugation (6,300 *g*, 4 °C, 5 min). Aliquots of the supernatant were adjusted to the appropriate CaCl_2_, EGTA or EDTA concentration and incubated with agitation for 15 min at 4 °C. Membranous (M) and cytoplasmic (C) fractions were separated by centrifugation at 100,000 *g*, 4 °C for 30 min. M and C fractions were subjected to SDS–PAGE followed by western blot analysis.

### Primary hippocampal cultures

Low-density cultures (∼150 cells per mm^2^) using the procedures described^24^ were used for expression studies (Fig. 1b). In brief, hippocampal cultures were established from 18-day-old fetal Wistar rat hippocampi. Primary astrocytes, used as feeder layer, were obtained from newborn rat cortical hemispheres.

High-density hippocampal primary neuronal cultures used for all other experiments were prepared as follows. The hippocampi were dissected from embryonic day (E) 18–19 rat or E16.5 mouse embryos. After dissection in Hank's balanced salt solution (HBSS; Invitrogen), hippocampi were washed in ice-cold HBSS. For dissociation, hippocampi were incubated for 12 min in trypsin at 37 °C followed by trituration in plating medium (MEM with GlutaMAX (Invitrogen), 0.6% glucose, 10% FBS (Gibco) and 1% penicillin/streptomycin (Invitrogen)). Neurons were plated at a density of 750 cells per mm^2^ on poly-L-lysine-coated glass slides. After 3 h, plating medium was replaced by culture medium (Neurobasal medium, supplemented with L-glutamine, B27 and penicillin/streptomycin (all from Invitrogen)).

Hippocampal cultures were transfected at DIV7, DIV14 or DIV19 with Lipofectamine 2000 (Invitrogen) following the manufacturer's instructions. cLTP and glutamate/glycine stimulation were induced as described previously^29^,^36^. Cultures were fixed with 4% PFA/PBS including 120 mM sucrose. Immunocytochemistry for Cofilin was performed as described previously^43^.

### Subcellular fractionation of rat brains

Fractionation was performed as described previously^65^ with minor modifications. Adult rat brains were homogenized in ice-cold buffer A (0.32 M sucrose, 1 mM NaHCO_3_, 1 mM MgCl_2_ and 0.5 mM CaCl_2_) using a glass/Teflon homogenizer. The homogenate was centrifuged at 1,400 *g* for 10 min at 4 °C to result in P1 (nuclei) and supernatant S1. The supernatant S1 was split into two aliquots. One was kept in high calcium and one was adjusted to a final concentration of 2 mM EDTA. In all the following steps, the two aliquots were treated separately. S1 aliquots were centrifuged at 13,800 *g* for 10 min at 4 °C, to yield P2 (crude synaptosomes) and S2. The S2 fractions were centrifuged at 100,000 *g* for 2 h, to obtain pellets consisting of microsomal and plasma membranes (P3). The two P2 pellets were resuspended in buffer A with or without 2 mM EDTA, respectively, and layered onto a discontinuous 0.85, 1.0 and 1.2 M sucrose density gradient in 1 mM NaHCO_3_ and centrifuged at 82,500 *g* for 2 h. The fractions located at the 1.0–1.2 M sucrose density interface were collected, resuspended in buffer B (0.32 M sucrose, 1 mM NaHCO_3_ and 0.5 mM CaCl_2_, (with or without 2 mM EDTA)) and centrifuged at 13,800 *g* for 10 min at 4 °C, to yield S4 and P4. The P4 fractions were resuspended and lysed in hypotonic buffer C (10 mM HEPES pH 8.0, protease inhibitor cocktail (Roche) and 0.5 mM CaCl_2_ (with or without 2 mM EDTA)) for 15 min at 4 °C. Synaptosomal cytoplasm fractions (S5) and SM were isolated by centrifugation at 13,800 *g* for 10 min at 4 °C. The SM pellets were resuspended in 10 mM HEPES pH 8.0, 150 mM NaCl, 2% Triton X-100 and 0.5 mM CaCl_2_ (with or without 2 mM EDTA) for 15 min at 4 °C. The following centrifugation (100,000 *g*, 30 min, 4 °C) resulted in PSD fractions and supernatants (S6). Protein concentrations in all the fractions were determined and equal amounts of protein were subjected to western blot analysis.

### Lipid raft isolation

Lipid raft isolation was performed as described previously^25^ and modified with minor modifications. After isolation of SMs, sample was adjusted to 45% sucrose. The 45% sucrose sample was overlaid with 12 ml of a linear sucrose gradient (35–5% in 50 mM Tris-HCl pH 7.5, 150 mM NaCl, 5 mM CaCl_2_ and protease inhibitor cocktail (Roche)). Floating lipid raft fractions were isolated by centrifugation at 100,000 *g* for 20 h at 4 °C.

### Co-immunoprecipitations

Mouse hippocampi were lysed in lysis buffer (20 mM Tris-HCl pH 8.0, 137 mM NaCl, 10% glycerol, 1% NP-40, 2 mM CaCl_2_ and protease inhibitor cocktail (Roche)) by a glass/Teflon homogenizer. Insoluble material was removed by centrifugation (16,000 *g*, 15 min, 4 °C). Total protein (0.5 mg) was incubated at 4 °C overnight with 4 μg Rac1 antibodies or non-immune mouse IgG as control.

For co-immunoprecipitations from COS7 cell lysates, cells were incubated with 2 mM dithiobis-succinimidyl propionate (Pierce) in PBS. After incubation on ice for 2 h, the reaction was stopped by adding 1 M Tris-HCl pH 7.5 to a final concentration of 20 mM followed by a further incubation of 15 min on ice. Cells were lysed in lysis buffer (50 mM Tris-HCl pH 7.5, 150 mM NaCl, 1% NP-40, 0.5% sodium deoxycholate and protease inhibitor cocktail (Roche)) for 20 min on ice and insoluble materials were removed by centrifugation at 10,000 *g* for 10 min at 4 °C. Antibodies (2 μg) were added to the supernatant and immune complexes were collected by incubating with Protein-G Sepharose. After washing with lysis buffer, bound proteins were eluted by SDS–PAGE loading buffer (5 min, 95 °C) and subjected to western blot analysis.

### Electrophysiology

Whole-cell patch-clamp recordings were performed from CA1 neurons of acute slices. Recordings were performed on sagittal hippocampal slices (400 μm for extracellular field recordings, 250–300 μm for whole-cell patch-clamp recordings) that were maintained in ACSF (119 mM NaCl, 1 mM NaH_2_PO_4_, 2.5 mM KCl, 2.5 mM CaCl_2_, 1.3 mM MgCl_2_, 11 mM D-glucose and 26.2 mM NaHCO_3_ constantly oxygenated with 95% O_2_/5% CO_2_). For whole-cell patch-clamp recordings, slices were cut in low calcium ACSF (0.125 mM CaCl_2_ and 3.3 mM MgCl_2_). Data were acquired with a MultiClamp 700B (Molecular Devices), low-pass filtered at 2 kHz and digitized at 10 kHz using a Digidata 1440 A interface (Molecular Devices) driven by pClamp 10.4 software. Miniature and evoked events were recorded using borosilicate glass pipettes (3–4 MΩ) filled with intracellular solution (135 mM CsMeSO_4_, 8 mM NaCl, 10 mM HEPES, 0.5 mM EGTA, 4 mM Mg-ATP, 0.3 mM Na-GTP and 5 mM Lidocaine-*N*-ethylbromid pH 7.3). For excitatory miniature postsynaptic current recording, the holding potential was set to −70 mV. For inhibitory miniature postsynaptic current recording, the holding potential was set to 0 mV. In both conditions, the postsynaptic current was recorded for 10 min in the presence of 0.5 μM tetrodotoxin. Traces were further analysed with the Mini Analysis Program v6 (Synaptosoft).

For LTD experiment, EPSCs were recorded at a holding potential of −70 mV in ACSF supplemented with picrotoxin (100 μM). EPSCs were evoked every 10 s by a stimulation electrode placed in the stratum radiatum. After 20 min of control recording, LTD was induced with a pairing stimulus of 1 Hz coupled with a postsynaptic depolarization to −40 mV for 5 min. The amplitude of each event was analysed using Clampfit 10.4 software (Molecular Devices). LTD recordings were performed on acute slices isolated from 25- to 35-day-old mice.

For extracellular field recordings, vibratome slices were transferred into oxygenated ACSF at 30 °C for 30 min. After this preincubation, slices were placed into an interface chamber perfused with oxygenated ACSF and equilibrated for at least 1 h. A Platinum/Iridium electrode (25 μm diameter) was used to stimulate the Schaffer collaterals and field potentials were recorded in the CA1 stratum radiatum with borosilicate glass pipettes (3–4 MΩ) filled with ACSF. fEPSPs were evoked by 0.33 Hz stimuli with a current that induced between 40 and 50% of maximal fEPSP. After recording of a stable baseline, LTP was induced by three trains of high-frequency stimulation (100 Hz for 1 s, with 240 s interval between trains). In jasplakinolide experiments, 0.2 μM jasplakinolide (Sigma-Aldrich, Cat. J4580) in ACSF was applied to slices as bath application via perfusion line.

### Analysis of spine density and morphology in CA1 neurons

To analyse spine density in the hippocampus, mice were intercrossed with mice expressing membrane-targeted GFP under the Thy1 promoter in a subset of CA1 neurons (line L15^31^). Six-week-old male littermate pairs were transcardially perfused with 4% PFA/PBS, brains were postfixed for 2 h in 4% PFA/PBS and cryoprotected in 30% sucrose. Brains were embedded in O.C.T. and cut into 80-μm-thick sections on a cryostat. Sections were mounted on glass slides and imaged with confocal microscopy (Leica SPE). Imaging, three-dimensional reconstruction of dendritic stretches and automated spine quantification were performed as previously described^66^. In brief, low-resolution images of CA1 neurons with 1 μm intervals along the *z* axis were taken. On these stacks, healthy-looking, secondary apical dendritic stretches that were clearly traceable up to the soma were selected and imaged at high resolution (0.08 μm *z* axis intervals, 1,024 × 1,024 pixels, 2.5 × digital zoom). After deconvolution (Huygens Deconvolution software), images were analysed with NeuronStudio software^67^. Detection of dendrites and spines were performed automatically followed by manual correction. For classification of spines, default NeuronStudio classification scheme was used^67^. The entire imaging and analysis procedure was done blinded to genotype.

### Imaging and image analysis

Epifluorescence microscopic pictures were obtained with a Leica DM5000B fluorescence microscope and analySIS software (Soft Imaging System). Confocal pictures used the Leica SPE or Leica SP5 confocal system using the software of the manufacturer. For time-lapse microscopy, glass slides with cultured neurons were transferred into a life imaging chamber (Ludin) filled with standard imaging solution (HBSS; Invitrogen) containing 10 mM HEPES pH 7.4, 2 mM glucose, 1.2 mM CaCl_2_ and 1 mM MgCl_2_). For glutamate stimulation, 100 μM glutamate and 10 μM glycine were added to the standard imaging solution. For NMDA stimulation, standard imaging solution without magnesium was supplemented with 10 μM NMDA (Tocris, Cat. 0114). Pictures shown in Supplementary Movie 1 and 2 were taken every 20–30 s for a total of up to 20 min with a Zeiss, Axiovert 135M Microscope. Confocal time-lapse microscopy (Fig. 2c and Supplementary Fig. 3b) used a Leica SP5 confocal system and a series of pictures (0.03 μm *z* axis intervals, 1,024 × 1,024 pixels, 2.5 × digital zoom) were taken every 30 s for up to 6 min after application of NMDA or glutamate. For quantification (Supplementary Fig. 3a,b), regions of interest within a spine and the corresponding neighbouring dendrite were pre-selected in the RFP channel and the Copine-6-GFP fluorescence intensity was determined in both regions. The ratio of the Copine-6-GFP fluorescence in the spines versus the dendrites was determined before and at the indicated time points after NMDA or glutamate application (Supplementary Fig. 3a,b).

For quantitative analysis of Copine-6-GFP, Copine-6^D167N^-GFP or GFP-Rac1 enrichment in spine versus dendrite in fixed preparations (Figs 2a, 4a, 6b and Supplementary Fig. 3c), a series of pictures (0.08 μm *z* axis intervals, 1,024 × 1,024 pixels, 2.5 × digital zoom) was taken and ten images were summed (*z*-stacked). A region of interest was outlined around the spine head and within the corresponding neighbouring dendrite and fluorescence intensities of GFP and RFP signals were measured with ImageJ software. The RFP signal in the spine was used to normalize to spine area; GFP and RFP signals in the dendrite were used to correct for differences in expression levels between neurons. In addition, GFP and RFP signals were corrected for background by subtracting the fluorescence signal in nearby, non-transfected neurons. ‘Relative GFP fluorescence in spine' was determined by calculating the ratio between (GFP_spine_/RFP_spine_) and (GFP_dendrite_/RFP_dendrite_).

Quantification of ‘relative Cofilin concentration in spine' was performed as described previously^43^ with some modifications. Intensity of the Cofilin signal in spines was normalized to the GFP signal in the spine and then divided by the GFP-normalized Cofilin signal in two regions of the dendrite connected to the spine (for illustration, see Supplementary Fig. 9a).

Measurements of spine head width and spine density in cultured hippocampal neurons were performed using ImageJ software. To measure spine density, only protrusions with clearly β-actin-positive heads were classified as spines. For the measurement of the spine head width, high-power pictures of randomly selected, secondary dendrites were used, mushroom-like spines were pre-selected and their width was determined. Imaging and image analysis was performed blinded to the experimental condition.

### Statistical analysis

Statistical analysis was performed using paired or unpaired Student's *t*-test for comparisons of two groups, one-way analysis of variance with Tukey's *post-hoc* test for comparisons of more than two groups, Kolmogorov–Smirnov test for the analysis of cumulative probabilities or two-way analysis of variance with Bonferroni *post-hoc* test as indicated in figure legends. All statistical tests were performed using Prism version 6 (GraphPad Software). Statistical significance was set at *P*<0.05. All data are presented as the mean±s.e.m., unless indicated otherwise in the figure legend. No statistical methods were used to pre-determine sample sizes but sample sizes are similar to those reported in the field. In imaging studies, cell selection was performed randomly after exclusion of unhealthy cells. Behaviour analysis and quantifications of microscopic data were performed by an investigator blinded to the experimental condition.

### Data availability

Full scans of immunoblots shown in main figures can be found in Supplementary Fig. 10. Relevant data that support the findings of this study are available from the corresponding author on request.

## Additional information

**How to cite this article**: Reinhard, J. R. *et al*. The calcium sensor Copine-6 regulates spine structural plasticity and learning and memory. *Nat. Commun.* 7:11613 doi: 10.1038/ncomms11613 (2016).

## Supplementary Material

Supplementary Figures Supplementary Figures 1-10 and Supplementary Reference

Supplementary Movie 1 Copine-6 translocates into spines after addition of NMDA. Time lapse microscopy of DIV14 hippocampal neurons expressing Copine-6-GFP (green) and cytosolic tdRFP (red). The movie starts immediately after the application of NMDA. The movie spans 14 minutes with 30 sec/frame.

Supplementary Movie 2 Distribution of GFP in hippocampal neurons is not affected by NMDA treatment. Time lapse microscopy of DIV14 hippocampal neurons expressing GFP (green) and cytosolic tdRFP (red). The movie starts immediately after the application of NMDA. The movie spans 20 minutes with 20 sec/frame.
