# Supplementary material for: The calcium sensor Copine-6 regulates spine structural plasticity and learning and memory
Source: Nat Commun. 2016 May 19;7:11613. doi: 10.1038/ncomms11613 (PMC4874034; doi:10.1038/ncomms11613)
Supplement: Supplementary Figures — 1-10 and Supplementary Reference [file ncomms11613-s1.pdf]

a

b

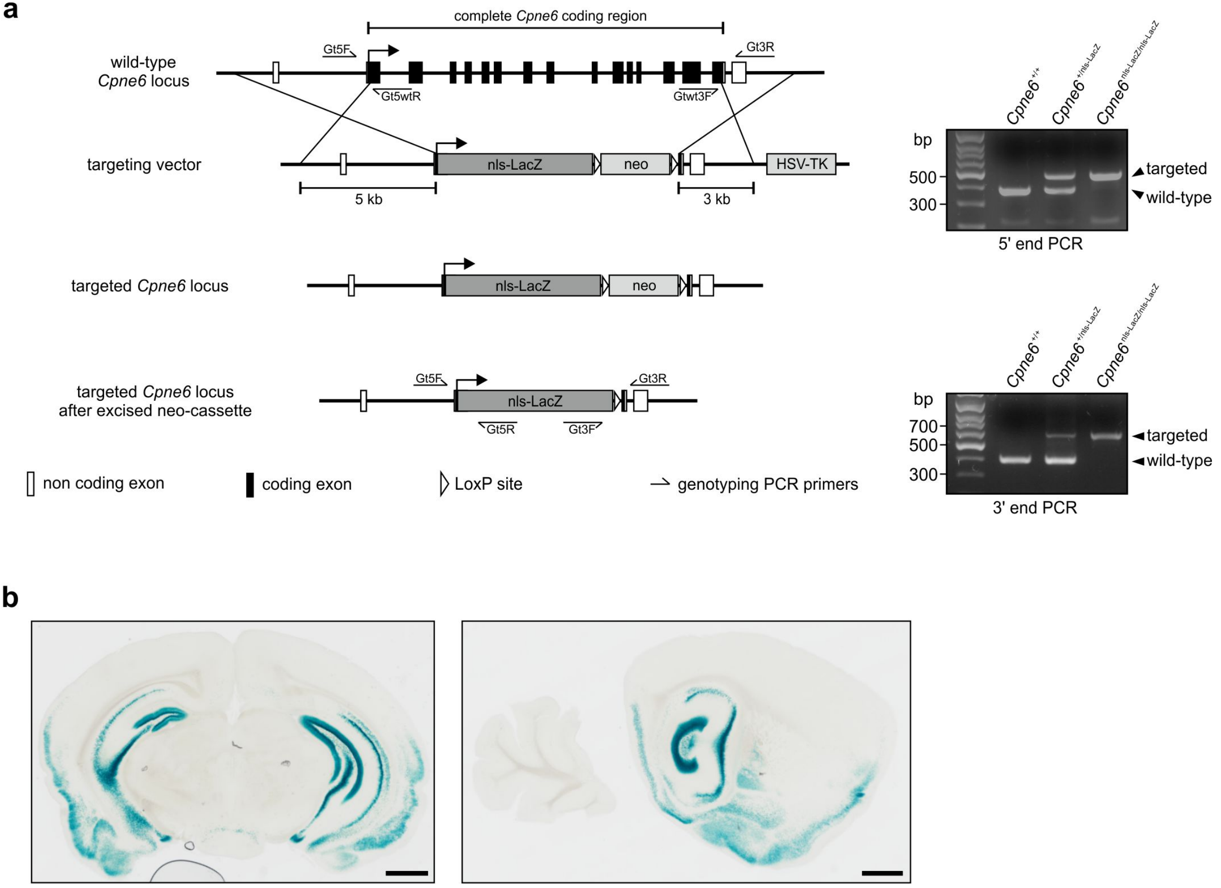


**Supplementary Figure 1**

**Generation of *Cpne6* knockin mice and regional expression of *Cpne6* in the adult mouse brain.**

(**a**) Targeting strategy to generate *Cpne6*$^{+/nls\text{-}LacZ}$ mice (left). All exons encoding Copine-6 were replaced by a cDNA encoding β-galactosidase preceded by a nuclear localization signal (nls-LacZ) by homologous recombination. Genotyping PCRs using lysates from biopsies of mice with the indicated genotypes confirm the presence of the appropriate alleles (right). Primers used were: 5’ end PCR: Gt5F, Gt5wtR and Gt5R; 3’ end PCR: Gt3F, Gt3wtR and Gt3R. (**b**) Coronal and sagittal brain sections of 6-week-old *Cpne6*$^{+/nls\text{-}LacZ}$ mice. *Cpne6*-driven nls-LacZ expression is detected by the blue β-gal staining in the nuclei. Scale bars, 1 mm.

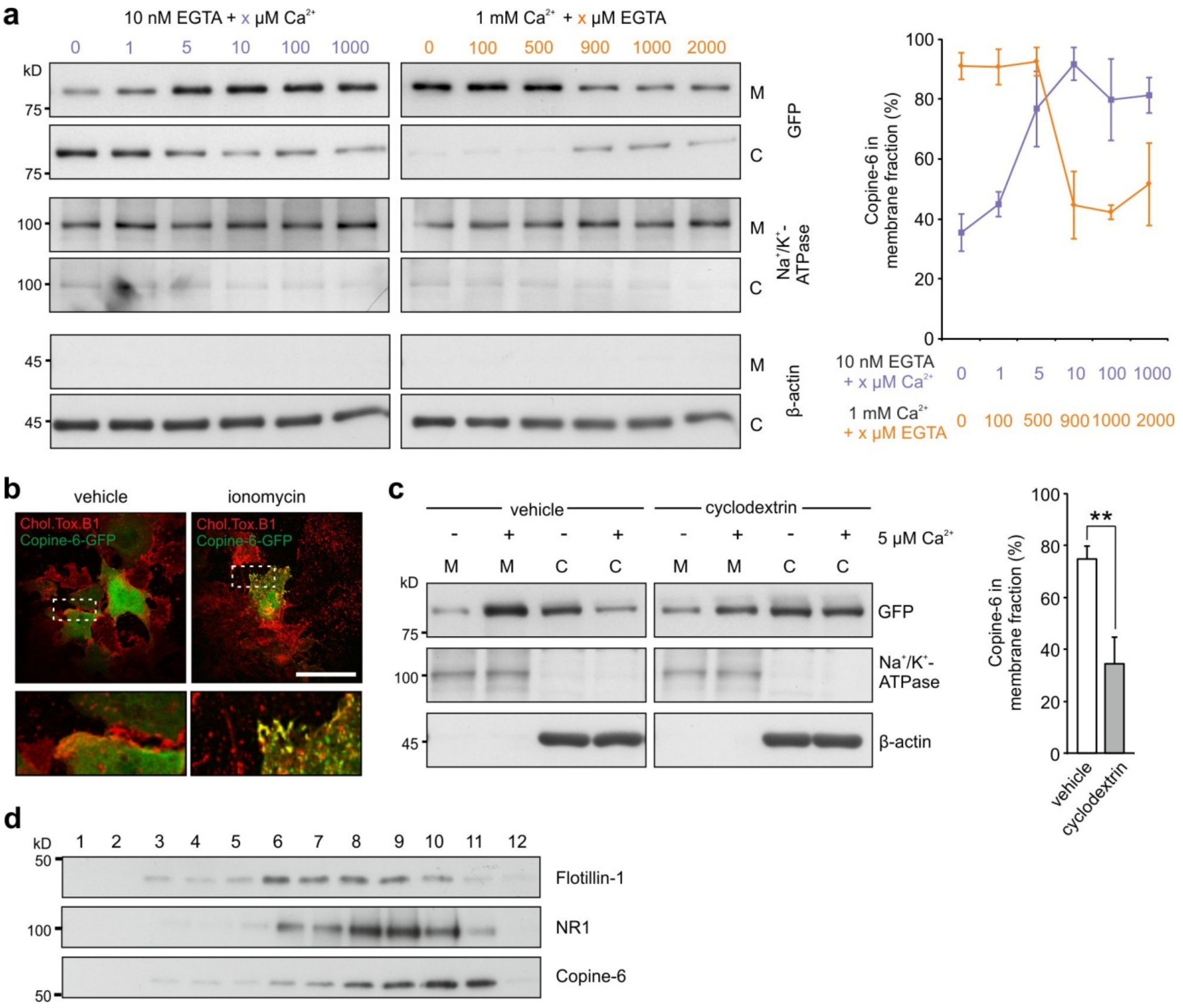


**Supplementary Figure 2**

**Calcium induces the reversible binding of Copine-6 to membranes and Copine-6 co-localizes and co-fractionates with lipid rafts.**

(**a**) COS7 cells, transfected with an expression construct encoding Copine-6-GFP, were lysed in the presence of 10 nM EGTA (left) or 1 mM $Ca^{2+}$ (middle). Increasing concentrations of $Ca^{2+}$ and EGTA, respectively, were added and lysates were separated into membranous (M) and cytosolic (C) fractions. The amount of Copine-6-GFP in those fractions was determined using antibodies to GFP (top row). Antibodies against the membrane protein $Na^{+}/K^{+}$-ATPase (middle row) and the cytosolic marker β-actin (bottom row) were used as fractionation and loading control. In the quantification (right), the amount of Copine-6-GFP in the membrane fraction is given as percentage of the total signal (C+M). Data are mean ± SEM. (**b**) COS7 cells expressing Copine-6-GFP (green) were stained with the lipid raft marker Alexa 647-labeled Cholera toxin B1 (red). In vehicle-treated cells, Copine-6-GFP appears cytosolic and does not co-localize with Cholera toxin B1. Incubation with the calcium ionophore ionomycin for 4 min induces membrane localization of Copine-6-GFP and co-localization with Cholera toxin B1 (yellow). Scale bar, 50 μm. (**c**) COS7 cells expressing Copine-6-GFP were treated with cyclodextrin or vehicle. Cell lysates were fractionated into membranous (M) and cytosolic fractions (C) in the presence or absence of calcium. The amount of Copine-6-GFP in each fraction was determined by Western blot analysis. $Na^{+}/K^{+}$-ATPase and β-actin were used as markers for membranous and cytosolic fractions, respectively, and as loading controls. Quantification of Western blot analysis of fractionations in presence of calcium is shown on the right. Data are mean ± SEM from n = 4 cultures. $^{**}P < 0.01$ by Student's t-test. (**d**) Adult rat brain membranes were fractionated in presence of calcium using a sucrose gradient. Western blot analysis for Flotillin-1, NMDA receptor subunit 1 (NR1) and Copine-6 was performed on 12 fractions (from top to bottom of the gradient).

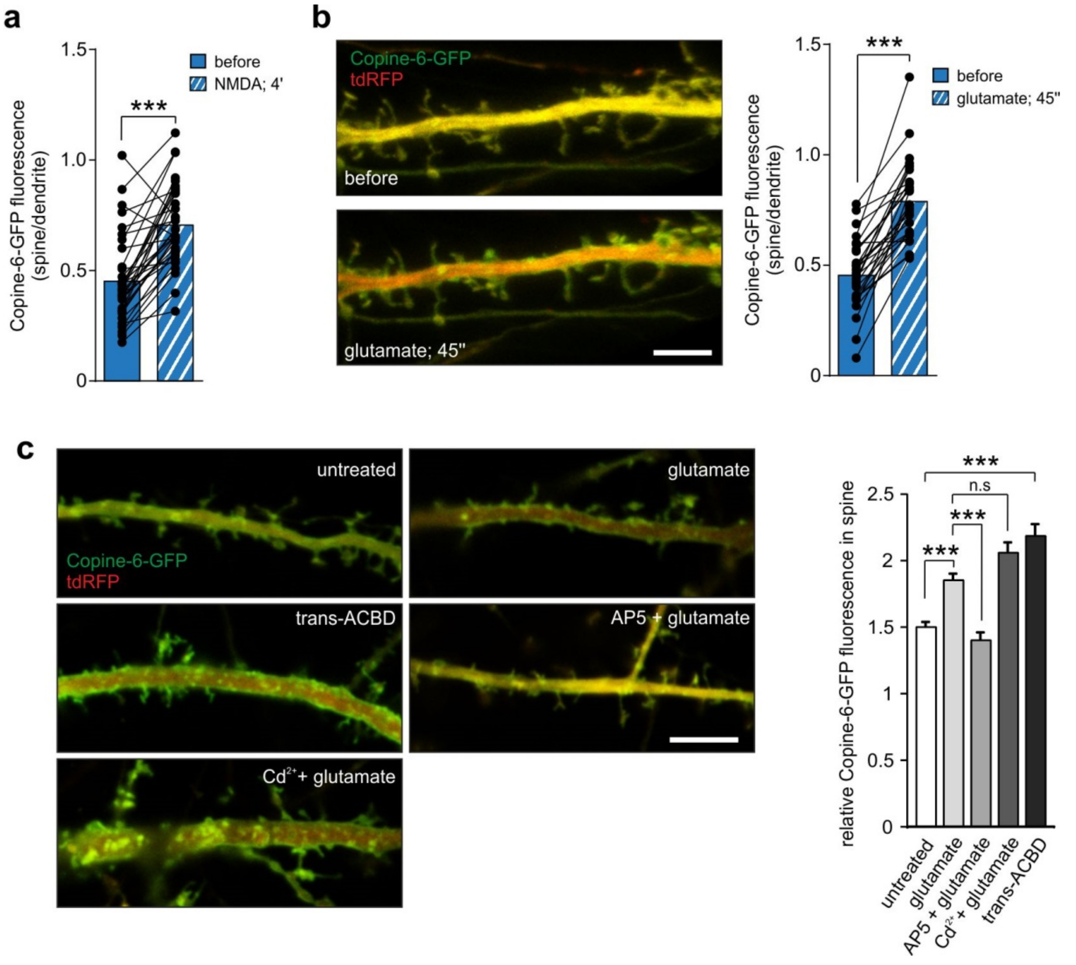


**Supplementary Figure 3**

**Copine-6 accumulates in spines upon NMDA receptor-mediated calcium influx.**

(**a**) Quantification of Copine-6 translocation shown in Figure 2c. Bars represent means from n = 29 spines from 6 neurons. ****P* < 0.001 by paired Student's t-test. (**b**) Time-lapse microscopy of DIV21 hippocampal neurons expressing Copine-6-GFP (green) and cytosolic tdRFP (red) before and 45 sec after glutamate application. Bars represent means from n = 29 spines from 6 neurons. ****P* < 0.001 by paired Student's t-test (**c**) DIV14 hippocampal neurons expressing Copine-6-GFP (green) and tdRFP (red) were treated for 5 min with 100 µM glutamate, 100 µM glutamate and 100 µM AP5, 100 µM glutamate and 100 µM $CdCl_2$ or 50 µM trans-ACBD. Addition of AP5 blocked Copine-6-GFP translocation upon glutamate application whereas cadmium ($Cd^{2+}$) did not affect it. Data are mean ± SEM from n = 48-60 spines from 8-10 neurons per condition. ****P* < 0.001; n.s. *P* > 0.05 by one-way ANOVA with Tukey's post-hoc test. Scale bar, 5 µm.

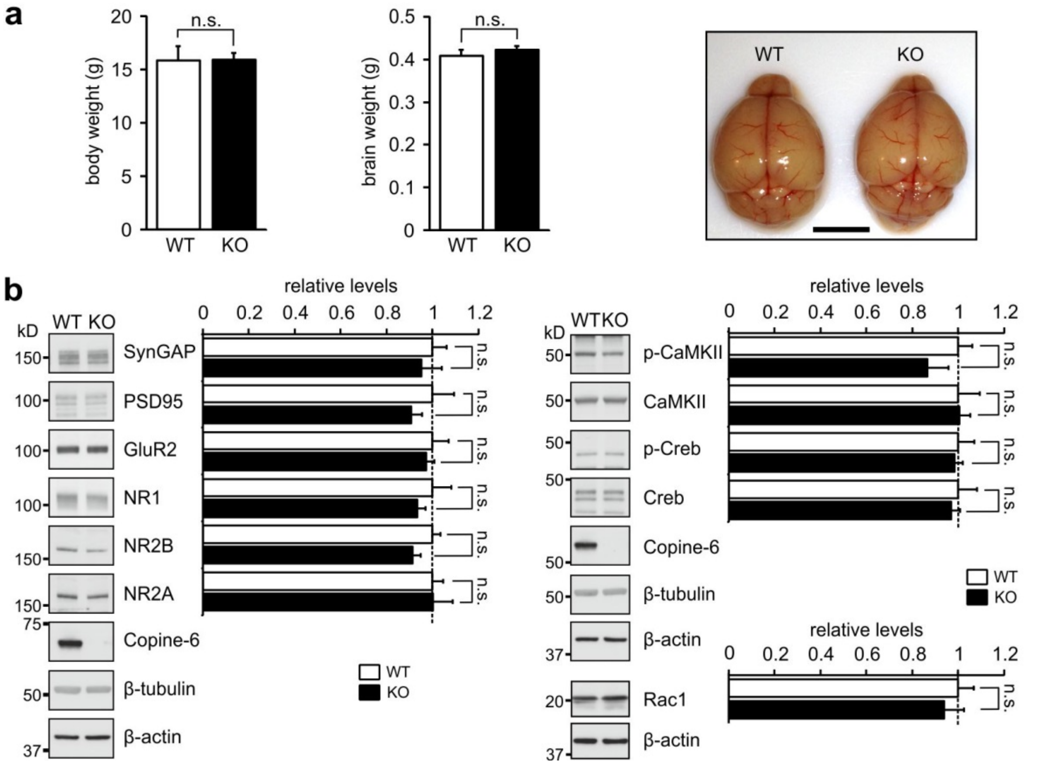


**Supplementary Figure 4**

**Biochemical analysis of synaptic proteins in *Cpne6* KO mice.**

(**a**) Quantification of body- and brain weight (left) and picture of dissected adult brains from wild-type (WT) and *Cpne6* KO mice. Data are mean ± SEM from n = 6 female mice (6-7-week-old) per genotype. n.s. $P > 0.05$ by Student's t-test. Scale bar, 5 mm. (**b**) Representative Western blots from hippocampal lysates of 6-week-old mice using antibodies directed against the proteins indicated. No significant change is observed in the amount of postsynaptic density proteins (left) or calcium signaling targets (right). Data are mean ± SEM from n = 5-12 mice per genotype (SynGAP: 12 WT and 8 KO mice, NR2A and NR2B: 12 WT and 9 KO mice, all others: 5 WT and 5 KO mice.). n.s. $P > 0.05$ by Student's t-test.

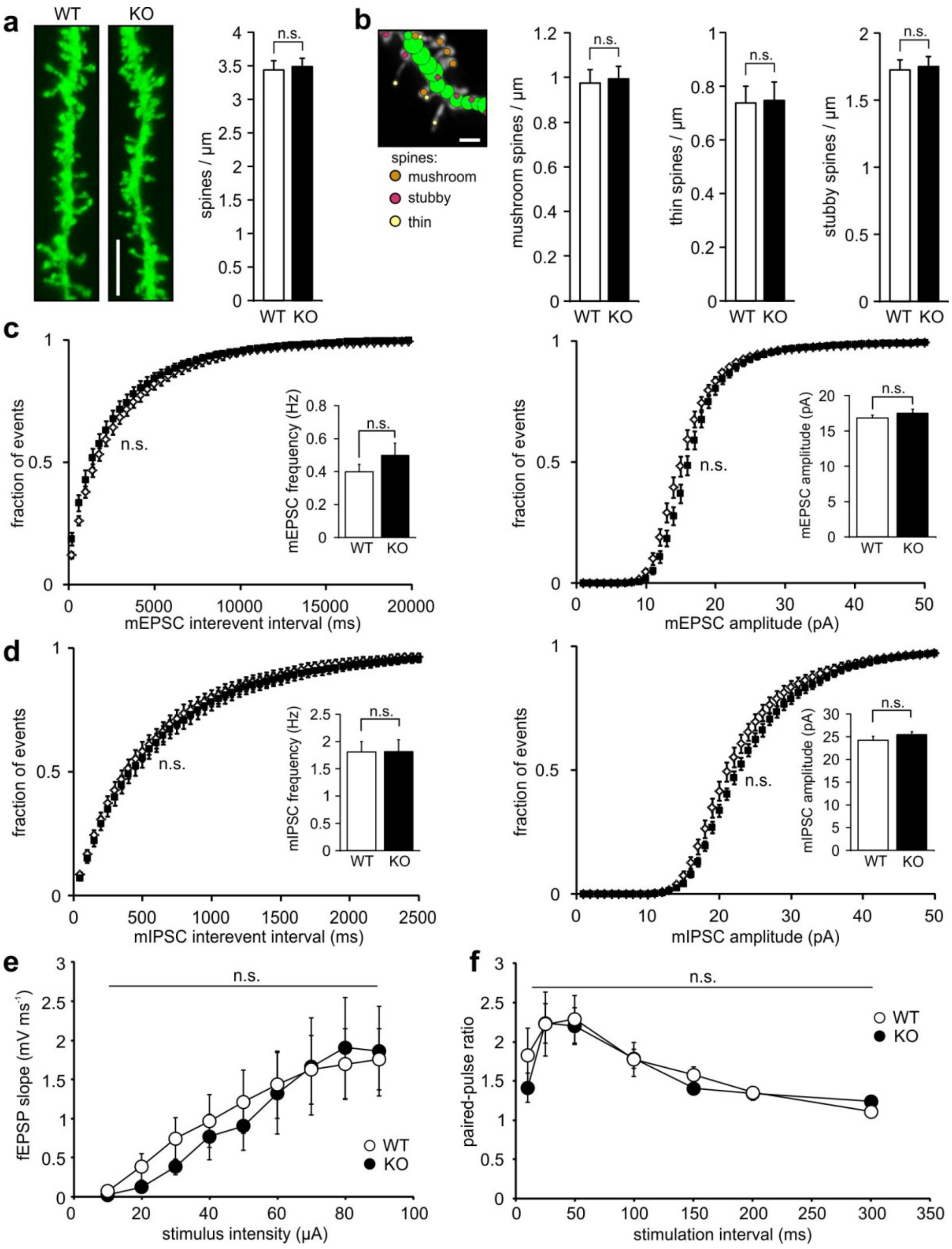


**Supplementary Figure 5**

***Cpne6* KO mice do not show alterations in spine density, morphology, mEPSCs and mIPSCs in CA1 neurons.**

(**a**) Representative secondary apical dendritic stretches of CA1 neurons in Thy1-mGFP mice on wild-type (WT) or *Cpne6* KO background (left) and quantification of spine density (right). Scale bar, 5 µm. n.s. $P > 0.05$ by Student's t-test. (**b**) Classification of spine types and respective density. Scale bar, 1 µm. n.s. $P > 0.05$ by Student's t-test. Data in **a** and **b** are mean ± SEM from n = 64 from four 6-week-old male mice per genotype (4 independent littermate pairs). 8 dendritic stretches from 8 different neurons per mouse were quantified. (**c** and **d**) Cumulative and mean frequency (left) and amplitude (right) of mEPSCs (in **c**) or mIPSCs (in **d**) of CA1 neurons from 6-week-old wild-type (WT) or *Cpne6* KO mice. Data in **c** and **d** are mean ± SEM from WT n = 18 cells and KO n = 17 cells (from 5 mice per genotype). For mean values: n.s. $P > 0.05$ by Student's t-test. For cumulative distributions: n.s. $P > 0.05$ by Kolmogorov-Smirnov test. (**e**) Input-output relationship measured in CA1 neurons by stimulation of Schaffer collaterals in acute hippocampal slices from 8-week-old wild-type (WT) or

*Cpne6* KO mice. Data are mean ± SEM from WT: n = 9 recordings per genotype (5 WT mice; 4 KO mice). n.s. $P > 0.05$ by two-way ANOVA with Bonferroni post-hoc test. (**f**) Paired-pulse ratio ($fEPSP_2$ slope to $fEPSP_1$ slope) measured in CA1 neurons by stimulation of Schaffer collaterals in acute hippocampal slices from 8-week-old wild-type (WT) or *Cpne6* KO. Data are mean ± SEM from WT: n = 9 recordings; KO: n = 11 recordings (5 WT mice; 5 KO mice). n.s. $P > 0.05$ by two-way ANOVA with Bonferroni post-hoc test.

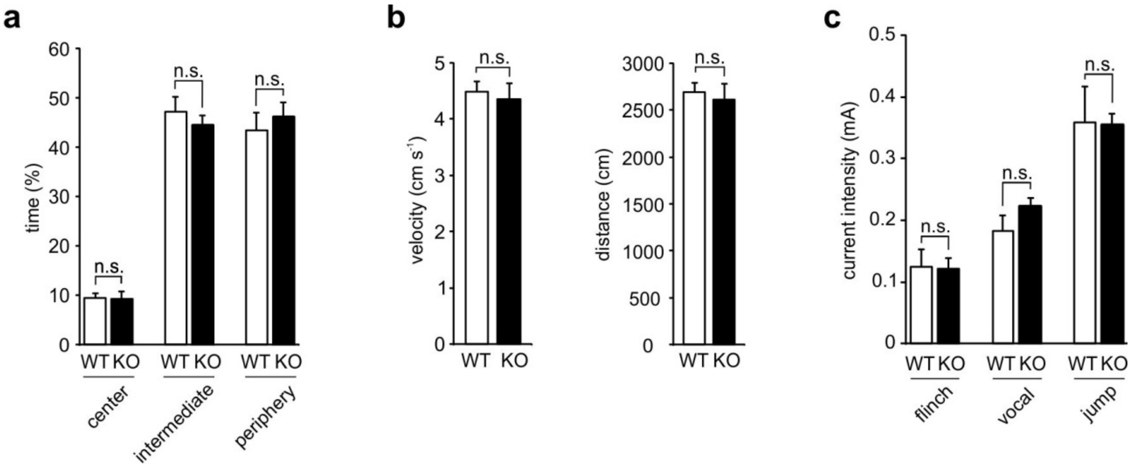


**Supplementary Figure 6**

**Open field behavior and foot-shock sensitivity are not altered in *Cpne6* KO mice.**

(**a**) Explorative behavior in a circular open field area of wild-type (WT) or *Cpne6* KO mice. n.s. $P > 0.05$ by two-way ANOVA and Bonferroni post-hoc test. (**b**) Velocity and distance of activities during open field test from wild-type (WT) or *Cpne6* KO mice. n.s. $P > 0.05$ by Student's t-test. Data in **a** and **b** are mean ± SEM from n = 12 male mice per genotype (6- to 10-week-old). (**c**) Foot-shock sensitivity of 8-week-old wild-type (WT) or *Cpne6* KO mice. Data are mean ± SEM from WT n = 6 male mice and KO n = 9 male mice. n.s. $P > 0.05$ by Student's t-test.

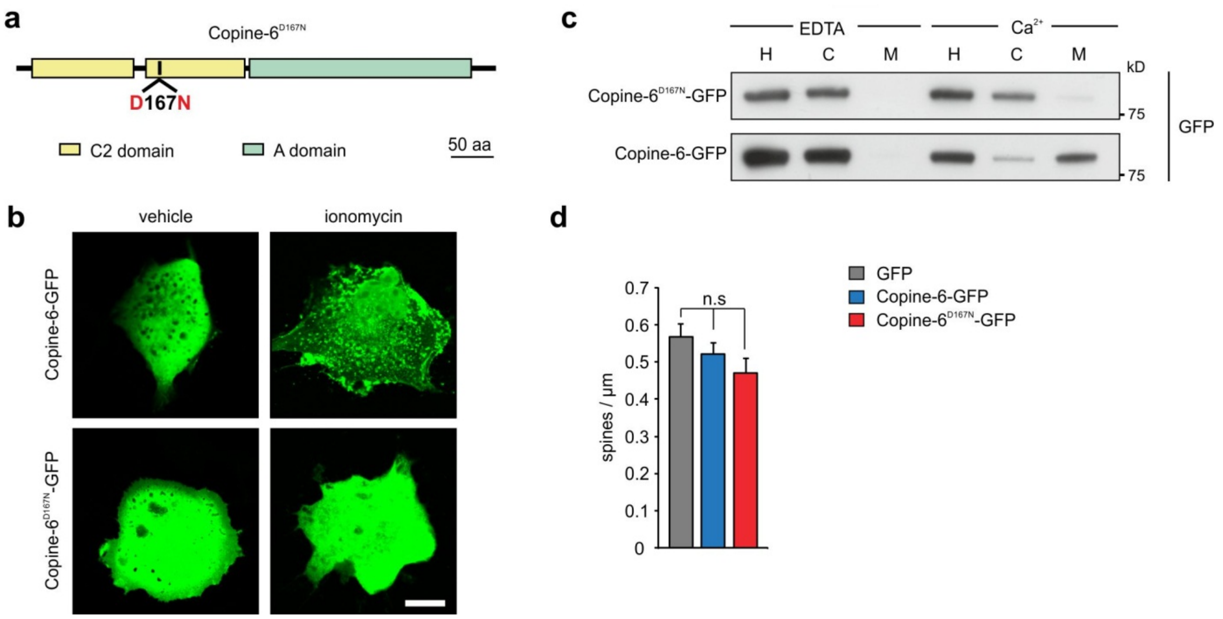


**Supplementary Figure 7**

**Generation and validation of calcium-insensitive Copine-6.**

(**a**) Schematic illustration of Copine-6 with a single amino acid mutation of Asp at position 167 to Asn (D167N). (**b**) COS7 cells transfected with Copine-6-GFP or Copine-6$^{D167N}$-GFP stimulated with ionomycin. Copine-6$^{D167N}$-GFP does not change its localization after ionomycin treatment. Scale bar, 20 µm. (**c**) Fractionation of COS7 cells expressing Copine-6$^{D167N}$-GFP or Copine-6-GFP in the presence of EDTA or calcium. Amount of the two proteins is similar in the cell homogenate (H). Copine-6$^{D167N}$-GFP remains in the cytosol (C) whereas Copine-6-GFP is enriched in membranous fractions (M) in the presence of calcium. (**d**) Primary hippocampal neurons were transfected at DIV14 with β-actin-tdRFP and indicated GFP constructs and analyzed at DIV21. Spine density is not significantly changed in primary dendrites. Representative pictures are shown in **Figure 4b**. Data are mean ± SEM from GFP and Copine-6-GFP: n = 11 neurons; Copine-6$^{D167N}$-GFP: 10 neurons. n.s. $P > 0.05$ by one-way ANOVA with Tukey's post-hoc test.

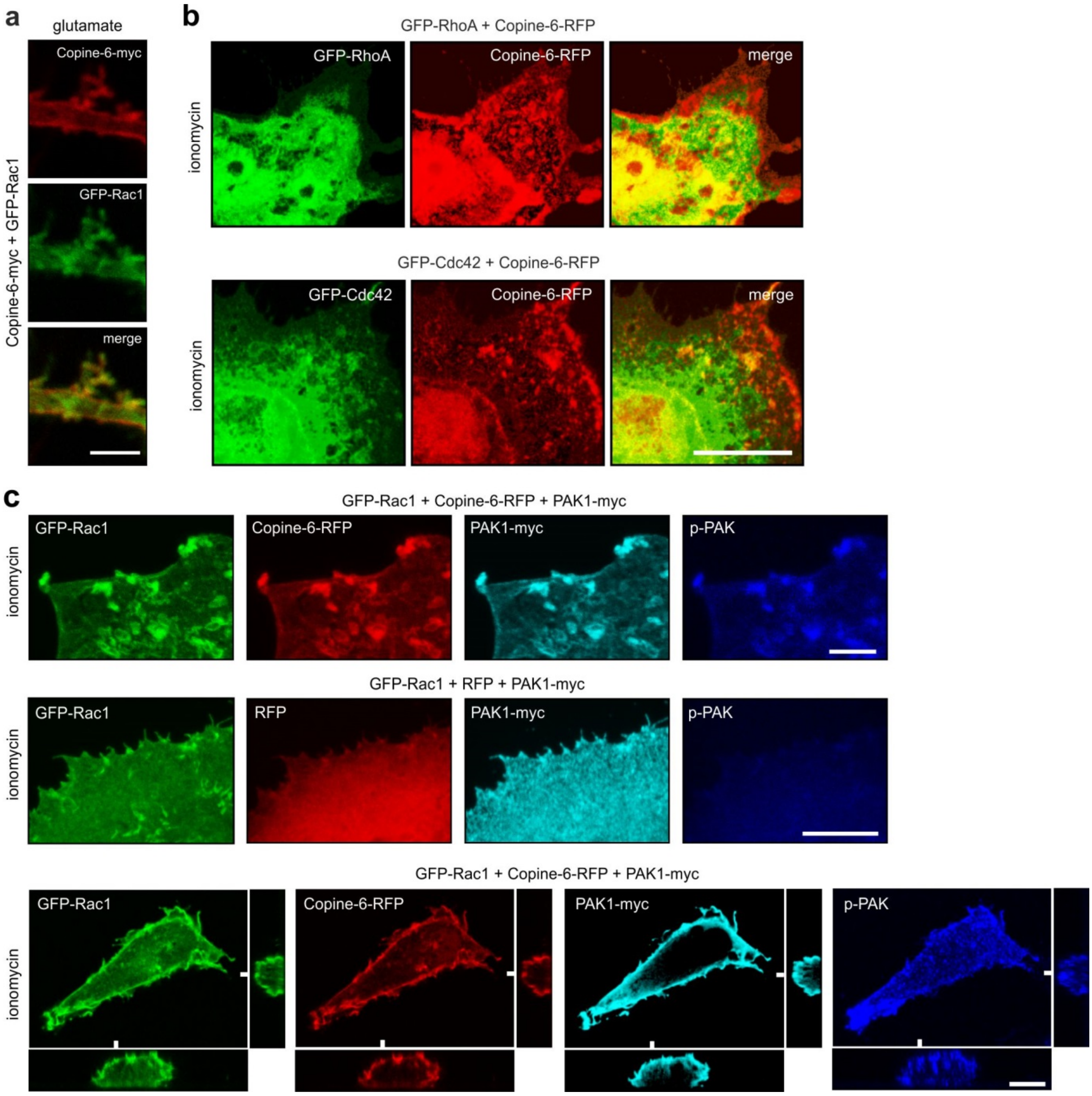


**Supplementary Figure 8**

**Calcium-mediated localization of Copine-6 to cell membranes does not affect RhoA or Cdc42 but co-recruits PAK1-myc together with GFP-Rac1.**

(**a**) DIV 21 hippocampal cultures, which were transfected at DIV19 with Copine-6-myc and GFP-Rac1, were stimulated for 5 min with 100 µM glutamate, fixed with PFA and stained for myc. Note that GFP-Rac1 (green) and Copine-6-myc (red) co-localize at spine membranes. Scale bar, 3 µm. (**b**) COS7 cells were co-transfected with Copine-6-RFP and GFP-RhoA (top) or GFP-Cdc42 (bottom). While treatment with ionomycin results in the localization of Copine-6-RFP to membranes, GFP-RhoA or GFP-Cdc42 are not co-recruited. Scale bar, 20 µm. (**c**) COS7 cells were triple transfected with expression constructs coding for GFP-Rac1, Copine-6-RFP (top, bottom) or RFP (middle), and PAK1-myc. Cells were treated with ionomycin for 4 min. In the presence of Copine-6-RFP, ionomycin-induced protein clusters are also positive for PAK1-myc in its activated, phosphorylated form (p-PAK1). Bottom: Confocal scan along the *x-z* and the *y-z* axes of a COS7 cell after ionomycin treatment. All proteins co-cluster at the plasma membrane. Scale bar, 5 µm (top, middle), 10 µm (bottom).

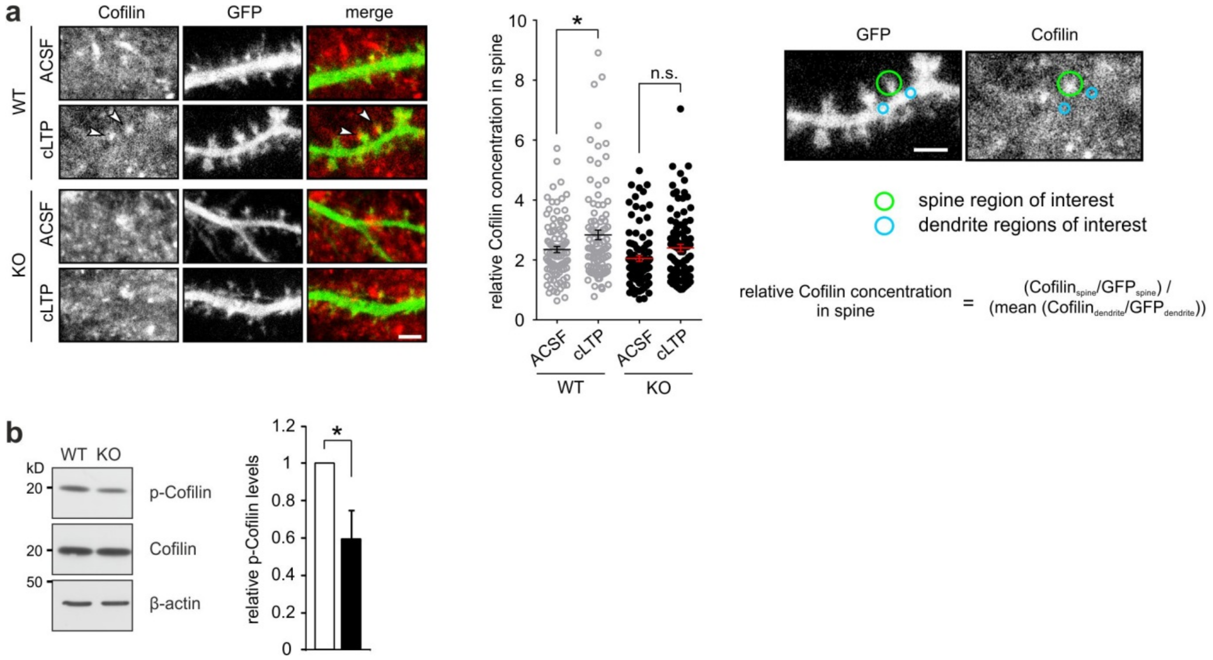


**Supplementary Figure 9**

**Copine-6 affects Cofilin localization and activity.**

(**a**) DIV21 hippocampal cultures from wild-type (WT) or *Cpne6* knockout (KO) mice, which had been transfected at DIV14 with an expression construct encoding GFP (for visualizing single neurons), were incubated with ACSF or cLTP-inducing ACSF (cLTP). After 10 min, cells were fixed and stained for endogenous Cofilin. In WT cultures, spines become strongly positive for Cofilin after cLTP induction (open arrowheads). Middle: quantification of the relative enrichment of Cofilin in spines of WT and KO cultures. While Cofilin concentration increases in spines of WT neurons by cLTP; there is no enrichment in KO cultures. Data are mean ± SEM from n = 96 spines per condition (4 spines per neuron from 24 neurons of each condition). *$P$ < 0.05; n.s. $P$ > 0.05 by one-way ANOVA with Tukey's post-hoc test. Right: For quantification of the relative Cofilin concentration in spines the method described elsewhere[1] was adapted as follows. Dendrites and spines were identified in the GFP channel and used to define regions of interest (spine: green circle; dendrite: blue circles). Mean intensities of Cofilin and GFP were measured in single optical sections. "Relative Cofilin concentration in spine", as was determined using the formula indicated. Note that this calculation determines the relative Cofilin concentration in spines irrespective of the spine volume. Scale bar, 2.5 µm. (**b**) Representative Western blots from DIV16 hippocampal cultures from wild-type (WT) or *Cpne6* knockout (KO) mice. Data are mean ± SEM from n = 6 independent cultures per genotype. *$P$ < 0.05 by Student's t-test.

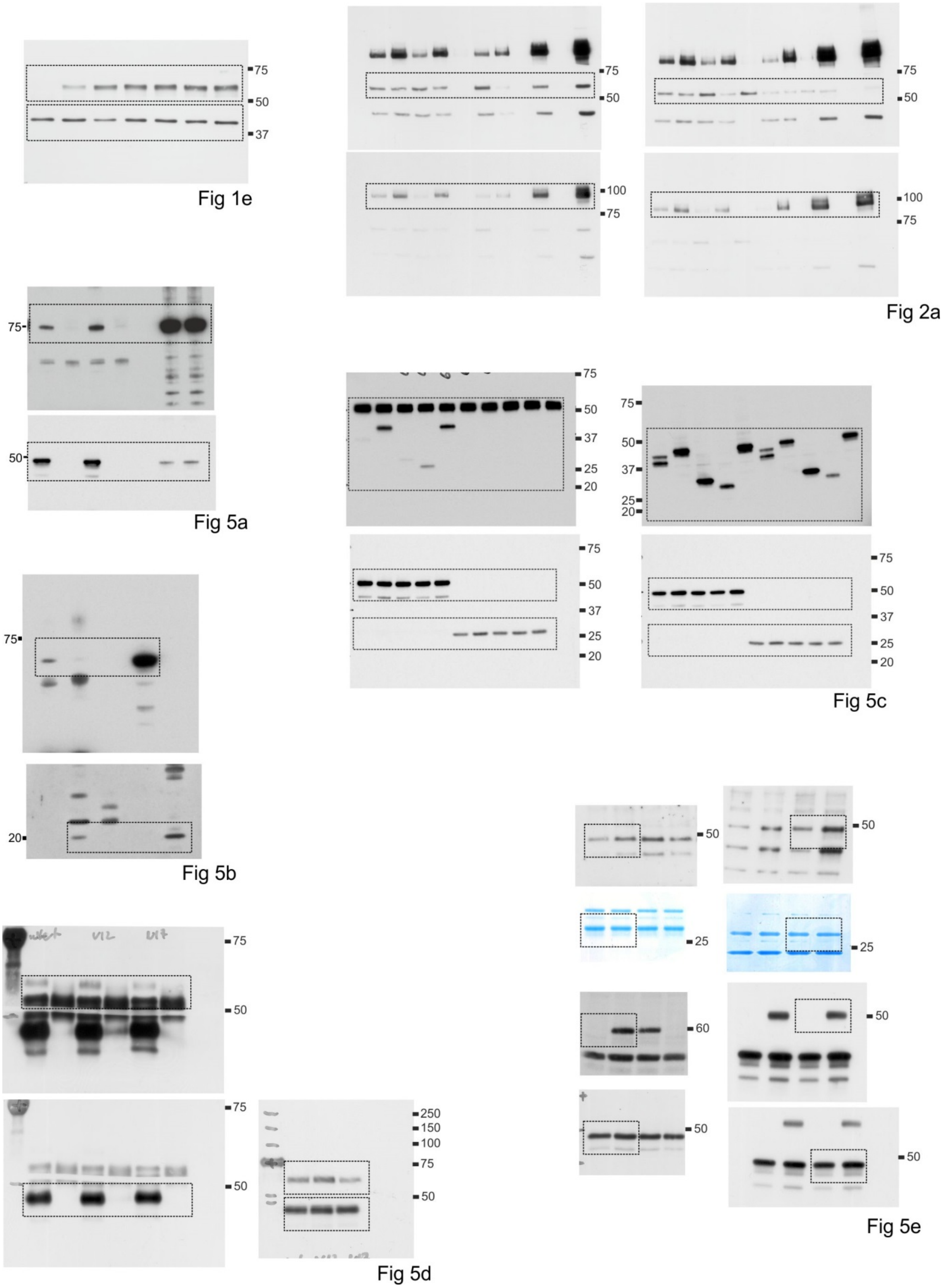


**Supplementary Figure 10**

**Full scans of immunoblots shown in main Figures**

**Reference**

1. Bosch, M., *et al.* Structural and molecular remodeling of dendritic spine substructures during long-term potentiation. *Neuron* **82**, 444-459 (2014).
